# Puerarin ameliorates acute lung injury by modulating NLRP3 inflammasome-induced pyroptosis

**DOI:** 10.1038/s41420-022-01137-8

**Published:** 2022-08-18

**Authors:** Dasheng Cai, Yue Zhao, Fang Yu

**Affiliations:** 1 Department of Anesthesiology, the First Hospital of China Medical University, Shenyang, PR China; 2 Department of Anesthesiology, Second Affiliated Hospital of Liaoning University of Traditional Chinese Medicine, Shenyang, PR China

**Keywords:** Trauma, Molecular biology

## Abstract

We commenced to analyze putative anti-pyroptosis effects of puerarin (PU) as mediated by the PP2A-HDAC1-NLRP3 pathway in acute lung injury (ALI). ALI animal and cell models were constructed, followed by treatment of PU. Then, the effect of HDAC1, PP2A, and NLRP3 on cell inflammation and pyroptosis was explored. The interaction between HDAC1 and PP2A as well as between PP2A and NLRP3 was analyzed. Our findings suggested that PU downregulated HDAC1 expression to alleviate symptoms of ALI. HDAC1 overexpression promoted inflammation induced by LPS, which reversed the inhibitory effect of PU on ALI. HDAC1 overexpression also decreased PP2A expression, suggesting that PP2A was involved in the effects of HDAC1 on LPS-induced inflammation. PP2A exerted inhibitory effects on NLRP3. Meanwhile, PU hindered the progression of ALI by silencing HDAC1 or overexpressing PP2A both in vivo and in vitro. Taken together, PU restrained pyroptosis of cells induced by NLRP3 inflammasome to abate ALI.

## Introduction

Acute lung injury (ALI) refers to a series of lung lesions resulting from multiple lung injuries, which may induce a severe lung condition, known as acute respiratory distress syndrome (ARDS), eventually leading to significant morbidity and death [1]. In addition, a significant proportion of patients who survive ALI/ARDS continue to suffer from physical, cognitive, and psychological dysfunction [2]. Thus, specific and efficient treatments and drugs for ALI warrant research. Pyroptosis refers to a pro-inflammatory process of cell death regulation that relies on the activity of inflammatory proteases, which are family members of cysteine-dependent aspartate-specific proteases [3]. The primary feature of pyroptosis is a rapidly ruptured cytoplasmic membrane leading to generation of intracellular contents and pro-inflammatory factors [4]. Furthermore, pyroptosis is detected in the ALI/ARDS mouse model and the LPS-induced macrophages [5].

Moreover, the inflammatory caspases may contribute to the pyroptosis [6]. NLRP3 is the key representative of inflammasome, the activation of which may induce Caspase 1-mediated proteolytic activation of the IL-18 and IL-1β, and trigger pyroptosis [7, 8]. As previously documented, NLRP3 inflammasome activation by the sensory receptor of cytosolic DNA is one of the crucial reasons causing lipopolysaccharide (LPS)-induced ALI [9]. Notably, attenuation of activation of the NLRP3 inflammasome is capable of preventing the progression of ALI [10]. Reduction of NLRP3 inflammasome activity in oxidized low-density lipoprotein-stimulated macrophages is associated with the upregulation of protein phosphatase 2 A (PP2A) [11]. PP2A activation can prevent inflammation and tissue injury in murine models of ALI [12]. Additionally, IKZF1 can modulate PP2A expression through recruitment of histone deacetylase 1 (HDAC1) [13]. Furthermore, HDAC1 is an essential epigenetic regulator and its inhibitor possesses suppressive role in the activation of pyroptosis [14, 15]. Therefore, we are curious about whether the HDAC1/IKZF1/PP2A axis is involved in regulating NLRP3 inflammasome-induced pyroptosis in ALI.

*Pueraria lobata* (Willd.) Ohwi is a traditional Chinese medicine, which is also called as Kudzu root [16]. It mainly possesses effects on fever, emesis, diarrhea, liver injury, cardiac dysfunctions, toxicosis, as well as weight loss [17]. Puerarin (PU) is the dominating ingredient with bioactivity extracted from the root of Gegen [18]. A recent study has also reported that PU can inhibit ALI through activating LXRα, which subsequently decreases LPS-mediated inflammatory response [19]. PU has been demonstrated to serve as an appealing therapeutic method to restrain diabetic osteoporosis due to its inhibiting effect on the HDAC1/HDAC3 signaling [20]. Importantly, PU can inhibit Aβ-induced NLRP3 inflammasome in retinal pigment epithelial cells [21]. Whether PU regulates NLRP3 inflammasome-induced pyroptosis in ALI through the HDAC1/IKZF1/PP2A axis is an interesting question.

Given the aforementioned evidence, this study intended to explore the potential roles of PU in mediating the process of pyroptosis involving HDAC1/PP2A, which eventually may attenuate the inflammatory in ALI and how PU regulates NLRP3 inflammasome-induced pyroptosis in ALI. This research may provide a therapeutic strategy to reduce inflammatory responses and lung injury in ALI.

## Results

### PU exerted inhibitory effect on ALI and HDAC1 was downregulated in the lung tissues of ALI mice

**Fig. 1:**
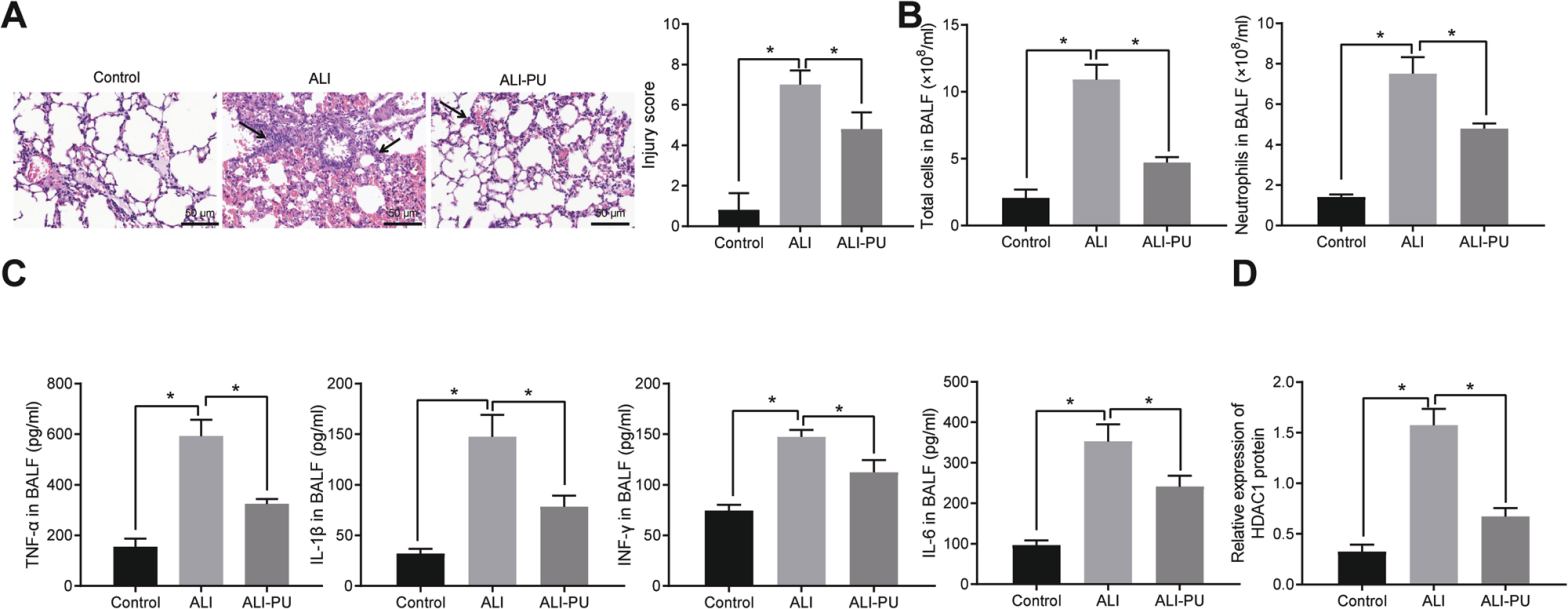
HDAC1 is expressed at a low level in lung tissues of PU-treated ALI mice. **A** Pathological changes of lung tissues in animal model, detected by HE staining (×200). **B** Total number of cells and neutrophils in BALF of animal model. **C** TNF-α, IL-1β, INF-γ and IL-6 levels in the BALF of animal model detected by ELISA. **D** HDAC1 expression in lung tissues of animal model detected by western blot analysis. *p < 0.05. *n* = 10.

As previously documented, PU has an inhibitory effect on ALI [19]. A mouse model of ALI was developed to assay the detailed molecular mechanism underlying the inhibitory effect of PU on ALI. As illustrated in Fig. 1A, ALI mouse showed significantly thickened alveolar septum, severely damaged lung tissues, and a large number of inflammatory cells infiltrated in the lung tissues, while ALI mice treated with PU showed a slightly widened alveolar septum, basically intact alveolar structure and reduced degree of inflammatory cell infiltration. In addition, the total number of cells and neutrophils as well as the levels of TNF-α, IL-1β, INF-γ, and IL-6 in bronchoalveolar lavage fluid (BALF) were increased in the ALI mice, while PU treatment led to the opposite trends (Fig. 1B, C).

Western blot analysis results (Fig. 1D, Supplementary Fig. 1A) depicted that the expression of HDAC1 in the lung tissues of mice in the ALI group was elevated while additional PU treatment reduced HDAC1 expression.

Hence, data above indicated that PU presented an inhibitory effect on ALI and HDAC1 was poorly expressed in the lung tissues of ALI mice.

### PU inhibited LPS-induced inflammation through HDAC1

**Fig. 2:**
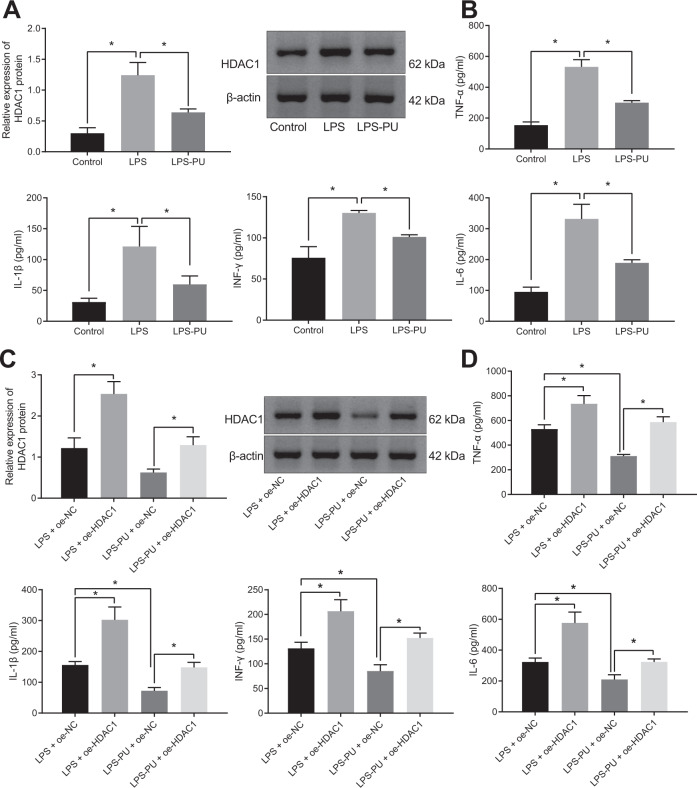
PU exerts inhibitory effect on LPS-induced inflammation by HDAC1. **A** HDAC1 protein expression in RAW 264.7 cells. **B** The level of inflammatory factors (TNF-α, IL-1β, INF-γ and IL-6) in RAW 264.7 cell supernatant assayed by ELISA. **C** The expression of HDAC1 in RAW 264.7 cells after transduction assayed by western blot analysis. **D** The level of lung inflammatory factors (TNF-α, IL-1β, INF-γ, and IL-6) in the transduced RAW 264.7 cell supernatant. *p < 0.05.

We then probed into the mechanism of inhibitory effect of PU on ALI, mouse macrophages RAW 264.7 were used to develop a cell model of inflammation by means of LPS. As displayed by Fig. 2A-C and Supplementary Fig. 1C, the expression of HDAC1 and the contents of TNF-α, IL-1β, INF-γ and IL-6 in the supernatant were increased in cells treated with LPS, all of which were reversed by further PU treatment.

Then, RAW 264.7 cells were treated with overexpressed HDAC1 (oe-HDAC1), followed by LPS or PU treatment. According to ELISA (Fig. 2D, Supplementary Fig. 1C), oe-HDAC1 significantly promoted LPS-induced inflammation as evidenced by upregulation of TNF-α, IL-1β, INF-γ, and IL-6. The addition of PU alleviated inflammation induced by LPS, which was counterweighed by overexpressing HDAC1.

In summary, PU was involved in the suppression of ALI by reducing HDAC1 expression.

### HDAC1 promoted LPS-induced cell inflammation by inhibiting PP2A

**Fig. 3:**
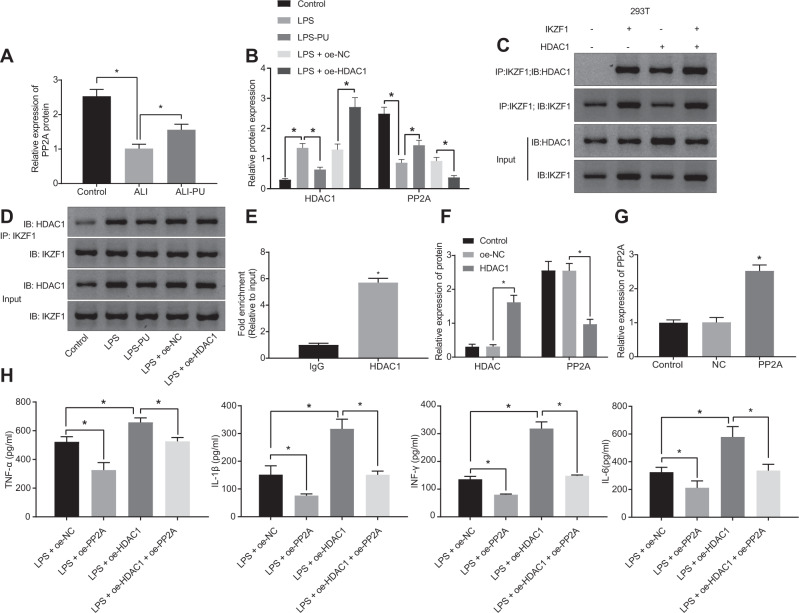
HDAC1 promotes LPS-induced cell inflammation through PP2A. **A** PP2A protein expression in lung tissues of animal models assayed by western blot analysis (*n* = 10). **B** The expression of HDAC1 and PP2A in RAW 264.7 cell models, checked by western blot analysis. **C** The interaction between HDAC1 and IKZF1 in 293 T cells, detected by Co-IP. **D** The interaction between HDAC1 and IKZF1 in RAW 264.7 cells detected by Co-IP. **E** The enrichment of HDAC1 in the PP2A HDAC1 bound to the promoters region, verified by ChIP. **F** The effect of HDAC1 on the expression of HDAC1 and PP2A in RAW 264.7 cells assessed by western blot analysis. **G** The effect of PP2A overexpression in RAW 264.7 cells checked by RT-qPCR. **H** The levels of lung inflammatory factors (TNF-α, IL-1β, INF-γ and IL-6) in RAW 264.7 cell supernatant after transduction evaluated by ELISA. *p < 0.05.

For assessing the role of HDAC1 in ALI was related to the expression of PP2A, animal and cell models were constructed. Western blot analysis indicated that the expression of PP2A was diminished in the ALI model but increased after further treatment of PU (Fig. 3A, Supplementary Fig. 1B). Moreover, in LPS-treated cells, elevated HDAC1 but reduced PP2A levels were observed, and further treatment with PU led to the opposite trends; in LPS-induced cells with oe-HDAC1 treatment, elevated HDAC1 but reduced PP2A were found relative to LPS + negative control for gene overexpression (oe-NC) treatment (Fig. 3B). Collectively, the expression of PP2A was reduced in ALI, while PU treatment promoted the expression of PP2A and overexpression of HDAC1 inhibited the expression of PP2A.

In order to confirm the mechanism of HDAC1 modulating PP2A expression, the interaction between HDAC1 and IKZF1 was tested by co-immunoprecipitation (Co-IP) experiments. Data presented obvious interaction between HDAC1 and IKZF1 in 293 T cells in vitro, and a strong interaction between the two was detected following concomitant overexpression of HDAC1 and IKZF1. In RAW 264.7 cells in vivo, interaction between HDAC1 and IKZF1 was also detected and the intensity of this interaction changed with the expression of HDAC1 protein. Specifically, PU treatment profoundly inhibited the protein expression of HDAC1 and the interaction between HDAC1 and IKZF1 was attenuated. However, overexpression of HDAC1 enhanced the interaction between HDAC1 and IKZF1 (Fig. 3C, D). Moreover, the results of chromatin immunoprecipitation (ChIP) assay verified that HDAC1 bound to the promoter region of PP2A (Fig. 3E). Furthermore, in cells transduced with oe-HDAC1, an increase in HDAC1 but a reduction in PP2A expression was found (Fig. 3F).

Additionally, whether the role of HDAC1 in LPS-induced inflammatory response was related to PP2A was explored with PP2A overexpressed in RAW 264.7 cells using lentivirus. RT-qPCR (Fig. 3G) depicted that the expression of PP2A in cells with oe-PP2A treatment was increased. In addition, ELISA results (Fig. 3H) noted that the contents of TNF-α, IL-1β, INF-γ and IL-6 were reduced in LPS-induced cell with oe-PP2A treatment alone while HDAC1 overexpression alone elevated the contents of TNF-α, IL-1β, INF-γ and IL-6. Moreover, PP2A overexpression significantly suppressed the contribution of HDAC1 overexpression on cell inflammation.

Collectively, HDAC1 promoted LPS-induced cellular inflammation by inhibiting PP2A.

### PP2A inhibited LPS-induced cell inflammation and pyroptosis by reducing NLRP3 inflammasome activation

**Fig. 4:**
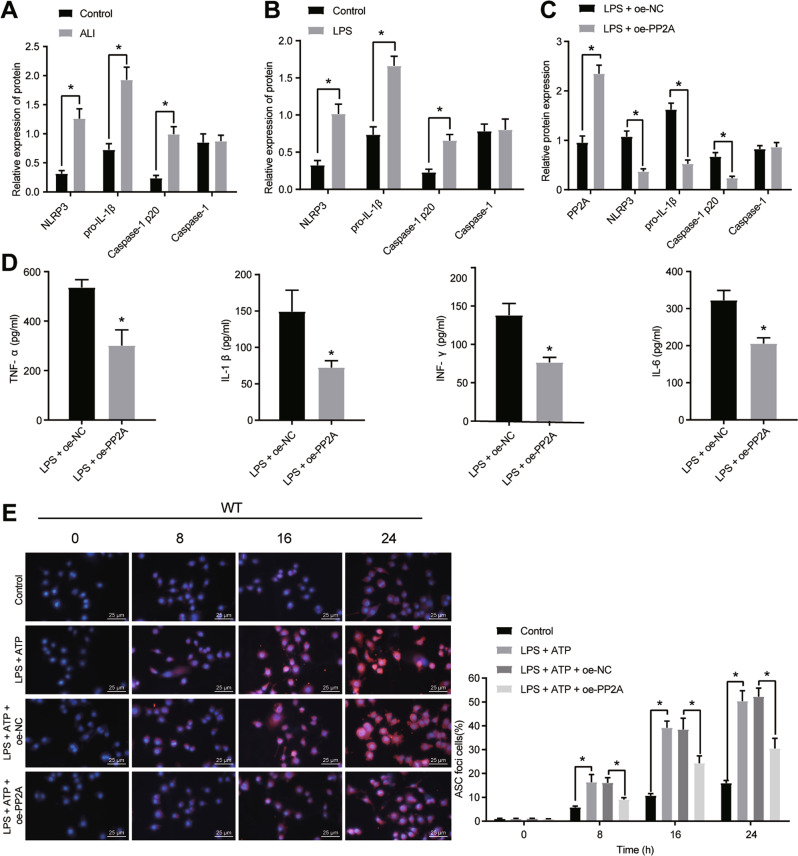
The inhibitory effect of overexpressed PP2A on LPS-induced cellular inflammation and pyroptosis. **A** The expression of NLRP3 inflammasome-related indicators in lung tissues of animal models (*n* = 10), detected by western blot analysis. **B** The expression of NLRP3 inflammasome-related indicators in cell models, detected by western blot analysis. **C** The expression of PP2A and NLRP3 inflammasome-related indicators after overexpression of PP2A, detected by western blot analysis. **D** The levels of lung inflammatory factors (TNF-α, IL-1β, INF-γ and IL-6) after overexpression of PP2A, detected by ELISA. **E** The formation of pyrosomes, detected by immunofluorescence (× 400). *p < 0.05.

Previous studies have documented that PP2A inhibits the NLRP3 inflammasome activation [12] and NLRP3 inflammasome promotes ALI [22]. Thus, the expression of NLRP3 inflammasome-related indicators in animal and cell models was first detected by Western blot analysis and the outcomes (Fig. 4A, B) indicated that ALI mouse lung tissues and LPS-induced cells showed elevated expression levels of NLRP3, pro-IL-1β, and Caspase-1 p20 protein, but Caspase-1 levels did not significantly change.

To discern that LPS-induced NLRP3 inflammasome activation was related to PP2A, RAW 264.7 cells were transfected with overexpressed PP2A. Western blot analysis was used to detect PP2A and NLRP3 inflammasome-related indicators and results revealed that (Fig. 4C) the expression of PP2A in LPS-treated cells upon PP2A treatment was increased, and the expression of NLRP3, pro-IL-1β, and Caspase-1 p20 protein was reduced, while Caspase-1 had no significant change. ELISA identified reduced levels of TNF-α, IL-1β, INF-γ, and IL-6 in LPS-treated cells upon PP2A treatment (Fig. 4D).

NLRP1-dependent apoptosis is capable of inducing ALI and morbidity in mice [23]. The formation of apoptosis-associated speck-like protein containing CARD (ASC) pyrosomes is a unique feature of caspase-1 induced pyrosomes [24]. Therefore, we detected the effect of PP2A on pyroptosis. RAW 264.7 cells were treated with LPS and ATP in vitro for 0 h, 8 h, 16 h, and 24 h followed by visualization of ASC by florescence-tagged ASC antibody and confocal microscopy. We found that the number of ASC foci positive cells was increased in the LPS-induced cells treated with ATP, while further addition of PP2A brought about contrary trends (Fig. 4E).

These results concluded that PP2A inhibited LPS-induced cell inflammation and pyroptosis by limiting the activation of NLRP3 inflammasome.

### Overexpression of HDAC1 or downregulation of PP2A in vivo inhibited the protective effect of PU in ALI models

**Fig. 5:**
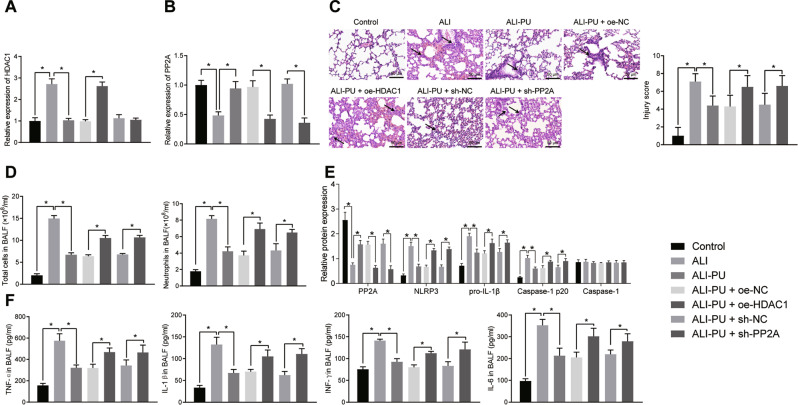
Overexpressed HDAC1 or silencing PP2A suppresses the protective effect of PU on ALI in mice. **A** The expression of HDAC1 in lung tissues of ALI mice after PU, oe-HDAC1 or sh-PP2A treatment, assayed by RT-qPCR. **B** The expression of PP2A in lung tissues of ALI mice after PU, oe-HDAC1 or sh-PP2A treatment, assayed by RT-qPCR. **C** The pathological changes in animal models and lung tissues after transfection (× 200), assessed by HE staining. **D** The total number of cells and the number of neutrophils in BALF in animal models after transfection. **E** The expression of PP2A and NLRP3 inflammasome-related indicators in lung tissues of animal models after transfection, assayed by Western blot analysis. **F** The levels of inflammatory factors (TNF-α, IL-1β, INF-γ and IL-6) in BALF of animal models after transfection, assayed by ELISA. * *p* < 0.05. *n* = 10.

For measuring the mechanism of HDAC1 or PP2A on ALI, a mouse model of ALI was constructed and then the ALI model was treated with overexpressed HDAC1 or silenced PP2A, followed by PU treatment. RT-qPCR (Fig. 5A, B) revealed that ALI mice and PU-treated ALI mice upon overexpressed HDAC1 treatment had elevated HDAC1 expression but reduced PP2A. However, relative to ALI mice, PU treatment led to decreased HDAC1 but increased PP2A expression. We also found short hairpin RNA (shRNA) against PP2A (sh-PP2A) treatment caused a reduction on PP2A expression in ALI mice.

Next, it was observed that upon HDAC1 overexpression or PP2A silencing in the PU treated ALI mice, the alveolar septum was significantly thickened and the lung tissues were severely damaged, a large number of inflammatory cells were infiltrated, and the total cell number, neutrophil number in BALF were increased (Fig. 5C, D). Detection of the PP2A and cell pyroptosis-related indicators by Western blot analysis demonstrated that upon HDAC1 overexpression or PP2A silencing in the PU treated ALI mice, the expression of PP2A was reduced and the expression of NLRP3, pro-IL-1β, and Caspase-1 p20 protein were increased, while the expression of Caspase-1 presented no significant changes (Fig. 5E, Supplementary Fig. 1D). These results suggested that overexpressed HDAC1 or silenced PP2A promoted cell pyroptosis in the lung tissues of mice.

Further, ELISA results displayed that after overexpression of HDAC1 or silencing of PP2A in the PU-treated ALI mice, elevated levels of TNF-α, IL-1β, INF-γ, and IL-6 were observed (Fig. 5F), indicating that overexpressed HDAC1 or silenced PP2A curbed the alleviation of inflammatory responses by PU in the lung tissues of mice.

These results suggested that upregulating HDAC1 or downregulating PP2A expression in vivo inhibited the protective effects of PU on ALI.

## Discussion

**Fig. 6:**
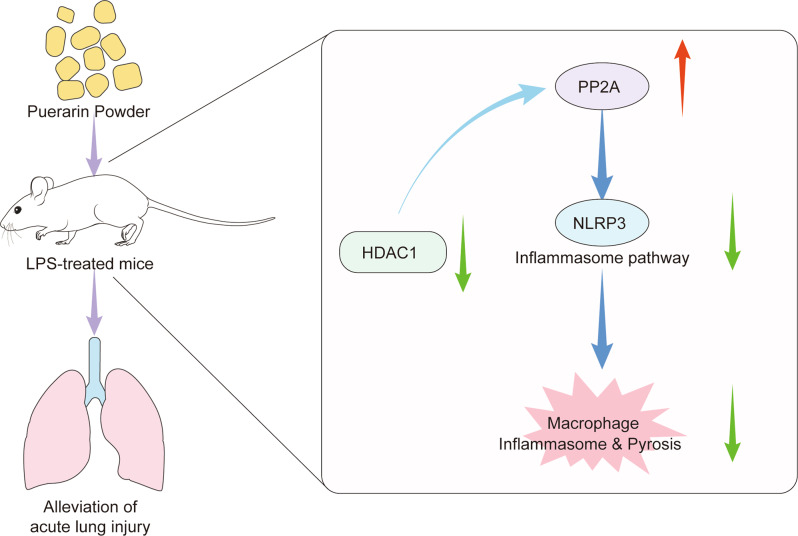
Schematic map of role of Puerarin in ALI. Puerarin ameliorated ALI by modulating NLRP3 inflammasome-induced pyroptosis.

Respiratory diseases and lung injuries are one of the primary causes of death all around the world [25]. An exaggerated inflammatory response is a critical characteristic of these diseases, including ALI [26]. Chinese medicine has long comprised the traditional approach for the treatment of ALI in China and has been found to be efficient [27]. Flavonoids possess a lot of biological functions and shows definite anti-inflammatory effects [28]. PU is the main bioactive flavone from traditional Chinese medicine [29]. Here, we attempted to uncover the putative role of PU in ALI. Our work emphasized that PU increased the expression of PP2A by downregulating HDAC1, leading to the inhibition of inflammation caused by the activation of the NLRP3 inflammasome (Fig. 6).

A previous study has demonstrated the inhibitory effects of PU on ALI [19, 30]. To verify the biological activity of PU on ALI, a mouse model with ALI was generated in our work and the anti-ALI effects of PU was verified in vivo. As reported, HDAC1 overexpression exacerbates inflammation and inhibiting HDAC1 prevents fructose-elicited inflammation in cells [20], which was in line with our finding that upregulation of HDAC1 in ALI was reduced by the treatment of PU. Moreover, LPS treatment induced HDAC1 expression in RAW 264.7 ALI cell model and overexpression of HDAC1 promoted the secretion of TNF-α, IL-1β, INF-γ, and IL-6. Proinflammatory cytokines TNF-α, IL-1β, INF-γ and IL-6 may be potential biomarkers for predicting the morbidity and mortality of ALI [1]. These results suggested a potential role of HDAC1 in ALI and PU treatment for ALI.

Furthermore, the mechanism of HDAC1 involving PU treatment for ALI was studied. Previous literature has shown that by interacting with IKZF1 and binding to the promoter of PP2A, HDAC1 can inhibit the expression of PP2A [13]. It is also reported that PP2A inhibits ALI [12]. Hence, the function of PP2A in PU-mediated ALI treatment was studied. PP2A is a dominating serine/threonine phosphatase and is extensively expressed in eukaryotic cells [31]. PP2A is of great importance to cell processes, such as cell metabolism, cell cycle, cell survival, and cell migration [32–35]. PP2A activation has been confirmed to abolish the inflammation in murine models of ALI [12]. Inhibition of PP2A contributes to NLRP3 inflammasome activation, resulting in IL-1β secretion and pyroptosis in hepatocytes [36]. In addition, metformin treatment can increase the activity of PP2A to alleviate the pro-inflammatory response in oxidized low-density lipoprotein-stimulated macrophages by blocking NLRP3 inflammasome activation through reducing NLRP3 [11]. The expression of PP2A was inhibited in the model of ALI and PP2A inhibits LPS-induced cellular inflammation and pyroptosis by inhibiting NLRP3 inflammatory bodies in this study.

As reported, PP2A inhibits the NLRP3 inflammasome and PP2A downregulation restores NLRP3 in macrophages [11]. The NLRP3 inflammasome has a crucial role in the process of diverse human inflammatory disorders, including atherosclerosis, diabetes, and Alzheimer’s disease [37]. The NLRP3 inflammasome was activated after LPS treatment in this study. It is reported NLRP3 inflammasome activation in infiltrating macrophages is observed in LPS-induced ALI [38]. IL-1β takes crucial and detrimental functions in the ALI development [39]. Notably, elevation of cleaved Caspase-1 is detected in lung tissues under the LPS challenge [40]. Caspase-1 autoproteolysis has been essential for NLRP3 inflammasome function [41–43]. Inflammasomes are able to form the molecular conditions that induce the Caspase-1 dimerization and autoproteolysis [44]. Likewise, we found that PU treatment or PP2A overexpression reduced Caspase-1 p20 protein expression by inhibiting the NLRP3 inflammasome in LPS-induced ALI. The formation of ASC coke is a unique feature of Caspase-1 induced pyroptosis [24]. It is known that NLRP1-dependent pyroptosis results in ALI and disease in mice [23]. Conclusively, PP2A inhibited LPS-induced cell inflammation and pyroptosis, which was modulated by the inhibition of NLRP3 inflammasome.

In conclusion, PU demonstrated anti-ALI activity by inhibiting the pyroptosis of cells, which was mediated by the regulation of the PP2A-HDAC1-NLRP3 inflammasome pathway. This research provided preliminary evidence suggesting a new mechanism for the PU-based treatment of ALI. In further studies, the dose-response relationship of PU effects on ALI should be addressed.

## Materials and methods

### Ethical statement

The current study was ratified by the Animal Ethics Committee of the First Hospital of China Medical University. The animal protocols were implemented in the light of the Guide for the Care and Use of Laboratory Animals published by the US National Institutes of Health.

### Construction of mouse models of ALI

C57BL/6 J mice (aged 6–8 weeks; weighed 18–22 g) were raised under a 12 h light/dark cycle each day (eat and drink freely). The mice were then randomized into the control, ALI and ALI-PU groups (ALI mice were treated with PU) with 10 mice in each group [45, 46]. The mice in the control group received no treatment. The mice in the ALI group were first anesthetized by intraperitoneal injection of 3% sodium pentobarbital, and a 5 mm median neck incision was made to expose the trachea. A microsyringe was used to instill LPS solution (2.5 mg/kg, L2630, Sigma-Aldrich, St. Louis, MO) from the trachea to the lungs within 1 min. The incision was then sterilized and sutured. The mice were then kept warm. As for the mice in the ALI-PU group, they were intraperitoneally injected with PU (30 mg/kg; P5555, Sigma-Aldrich) intraperitoneally 1 h before the LPS injection.

Meantime, to assess the role of HDAC1 and PP2A in ALI, mice were taken out and injected intraperitoneally with lentivirus-based oe-HDAC1 and sh-PP2A (5 × 10^8^ pfu/100 μL) 6 days before ALI-PU treatment. Synthesis of lentivirus-mediated oe-HDAC1 and sh-PP2A was performed by HanBio Technology (Shanghai, China). The mice were divided into the ALI-PU + oe-NC group (ALI mice were treated with PU and LPS after injection of lentivirus-mediated oe-NC), the ALI-PU + oe-HDAC1 group (ALI mice were treated with PU and LPS treatment after injection of lentivirus overexpressing HDAC1), the ALI-PU + sh-NC group (ALI mice were treated with PU and LPS treatment after injection of shRNA against NC lentivirus), and the ALI-PU + sh-PP2A group (ALI mice were treated with PU and LPS treatment after injection of shRNA against PP2A lentivirus) with 10 mice in each group.

All mice were euthanized 12 h after LPS treatment and the BALF and lung tissues were harvested for subsequent experimentations.

### BALF collection

Mice were euthanized. After exposure of the trachea, tracheal intubation was performed. Phosphate buffer saline (PBS)-ethylenediamine tetraacetic acid (1 mL) was flushed into the bilateral bronchoalveoli of mice through tracheal intubation by a syringe (1 mL). BALF was collected after 3 washes and this collection was repeated for 3 times. Then, the collected BALFs were mixed and centrifuged at 3000 rpm and 4 °C for 10 min. BALF supernatant and precipitate were stored separately.

### Inflammatory cell count

BALF pellet was lysed with erythrocyte lysis (1 mL) and centrifuged at 3000 rpm and 4 °C for 10 min with the supernatant discarded. The pellet was resuspended in 1 mL of PBS. After mixing, 10 μL of the mixture was pipetted onto the cell counting plate. Cells were stained by Wright-Gimsa staining and the inflammatory cells were classified and counted under a light microscope [47].

### Hematoxylin-eosin (HE) staining

Tissues from the lower right lung of mice were prepared into paraffin-embedded sections which were stained with hematoxylin for 4 min and counterstained with eosin for 2 min. The sections were observed under an optical microscope (CSW-PH50, Shenzhen Christie Optical Instruments Co., Ltd. Shenzhen, China) with histopathological changes evaluated and graded in a blind manner utilizing an arbitrary grading scale. The resultant pulmonary parameters were assessed, including hemorrhage, alveolar neutrophil infiltration, infiltration, and edema of interstitial and perivascular cells, as well as alveolar epithelial necrosis [48].

### ELISA

According to the standard procedures on the instructions, concentrations of TNF-α, IL-1β, IL-6, and IFN-γ in the supernatant were measured using ELISA kits (KS10484, KS10929, KS18212, and KS18210; all form Keshun Biotechnology, Shanghai, China).

### Cell culture

RAW 264.7 macrophages (CL-0190, Procell Life Science & Technology Co., Ltd., Wuhan, Hubei, China) derived from the mouse blood were cultured with a high-glucose Dulbecco’s modified Eagle’s medium (DMEM) replenished with 10% fetal bovine serum, 100 U/mL penicillin and 100 mg/mL streptomycin in a 5% CO_2_ incubator at 37 °C. RAW 264.7 cells were randomly divided into 3 groups and placed in sterile centrifuge tubes, which were labeled as control (routinely cultured without extra treatment), LPS (treated with 0.5 μg/mL LPS) and LPS-PU (treated with 0.5 μg/mL LPS and 20 μM PU) with 5 × 10^5^ cells in each group. Then, cells were cultured in a complete medium (5 mL) for another 24 h. For detection of pyrophysis, cells were treated with 0.5 μg/mL LPS for 0–24 h and then with 5 mM ATP (A6559, Sigma-Aldrich) for 30 min.

### Immunofluorescence

RAW 264.7 cells (1000 cells) were incubated on a cover glass coated with culture medium, and washed thrice with pre-warmed PBS for 10 min each when reaching 95–100% confluence. RAW 264.7 cells were then fixed with 4% paraformaldehyde for 20 min, immersed in PBS (containing 0.1% Triton X-100) for 10 min, blocked in PBS with 0.1% Tween-20 replenished with 3% bovine serum protein for 2 h and incubated with rabbit polyclonal antibody against TMS1/ASC ASC-speck formation; ab227502, 1: 100, Abcam Inc., Cambridge, UK) at 4 °C overnight. After that, the cells were incubated with Alexa-Fluor 555 conjugated donkey anti-rabbit IgG (ab150074, 1: 1000, Abcam) for 1 h. Subsequently, the nuclei were stained with 4′,6-diamidino-2-phenylindole (D9542, MERCK). Following this, the cells were observed under a Cellnsight CX7 LZR confocal microscope (CX7 LZR, Thermo Fisher Scientific Inc., Waltham, MA).

### Construction of lentivirus

Primers for PCR were designed based on the mouse HDAC1, PP2A and IKZF1 gene sequences. The primer sequences were: HDAC1 (forward: 5′-GAGCAAGATGGCGCAGACTC-3′, reverse: 5′-CTGGTCCCTGGGGACGTTAT-3′), PP2A (forward: 5′-ATTACAGAAAGCCGAGTCCCG-3′, reverse: 5′-GCGTCAGCATGCAATGAACT-3′) and IKZF1 (forward: 5′-CCAGGATCATTCTTGGCCCC-3′, reverse: 5′-AATGCTGCCTGCAAATCCAC-3′). HDAC1, PP2A and IKZF1 overexpression vectors were developed employing pLV-EGFP-N lentivirus, namely pLV-HDAC1, pLV-PP2A and pLV-IKZF1. Meanwhile, PP2A was knocked down using pSIH1-H1-copGFP lentivirus vector (shRNA sequence: 5′-CGACGAGTGTTTAAGGAAATA-3′), being pSIHI-PP2A. pLV-HDAC1, pLV-PP2A, pLV-IKZF1, and pSIHI-PP2A were packaged and purified by HanBio Technology (Shanghai).

### Cell transduction and grouping

One day before transduction, RAW 264.7 cells were trypsinized, counted, seeded into 6 wells (2 × 10^5^ cells/well) and then cultured in DMEM in an incubator with 5% CO_2_ at 37 °C. Upon 30–50% confluence, the original medium was renewed and the serum-free medium (1 mL) containing antibacterial agents was added with 2 × 10^6^ TU lentivirus and 5 μg Polybrene (Sigma-Aldrich) for transduction in an incubator with 5% CO_2_ at 37 °C. The second day, the medium containing lentivirus was removed and 2 mL fresh complete was added for overnight culture. The transduction efficiency was detected under a fluorescence microscope and the transduction rate was calculated: transduction rate=the area of blue-stained cells on the cross section/the area of all cells × 100%. After 48 h of transduction, each well was added with 1 μg/mL puromycin to screen out the stably transduced cells.

Subsequently, stably transduced RAW 264.7 cells were treated with oe-NC (transduced with lentivirus empty vector), oe-HDAC1 (transduced with lentivirus-mediated HDAC1 overexpression vector) oe-PP2A (transduced with lentivirus-mediated PP2A overexpression vector), oe-IKZF1 (transduced with lentivirus-mediated IKZF1 overexpression vector), LPS or/and both LPS and PU (LPS-PU), alone or in combination. The sequences for overexpression vectors are summarized in Supplementary Table 1.

### RT-qPCR

TRIzol-extracted total RNA was prepared. RT-qPCR was conducted using SYBRGreen fluorescent dye (RR091A, TAKARA, Japan) on an ABI 7500 qPCR instrument (Applied Biosystems, Foster City, CA). Sequences for all primer pairs (Invitrogen) are summarized in Supplementary Table 2. The relative quantification method was used and β-actin was set as the internal reference. The 2^-△△Ct^ method was used to calculate the relative transcription level of target genes.

### Western blot analysis

Total protein was extracted, electrophoresed and then electroblotted to a polyvinylidene fluoride membrane which was incubated with primary antibodies against HDAC1 (ab53091, 1: 1000, Abcam), PP2A (ab32065, 1: 500, Abcam), NLRP3 (ab263899, 1: 1000, Abcam), pro-IL-1β (ab234437, 1: 1000, Abcam), Caspase-1 (ab138483, 1: 1000, Abcam), Caspase-1 p20 (sc-398715, 1: 2000, Santa Cruz Biotechnology, CA) and β-actin (ab8226, 1: 5000, Abcam, internal reference) at 4 °C overnight. Horseradish peroxidase-labeled secondary antibody of goat anti-rabbit IgG (ab97051, 1: 2000, Abcam) was added for another 1-h of culturing with membrane. The membrane was immersed in an enhanced chemiluminescence reaction solution (BM101, Biomiga) for development.

### Co-IP

The interaction between HDAC1 and IKZF1 proteins was verified as follows. The 293 T cells (CL-0005, Procell, culture conditions were the same as RAW 264.7 cells) were transfected with oe-NC, oe-HDAC1, oe-IKZF1 or co-transfected with oe-HDAC1 and oe-IKZF1 employing Lipofectamine 2000 reagent (12566014, Thermo Fisher Scientific) for 48 h. Next, cells were lysed with 300 μL cell lysate containing protease inhibitor. The supernatant was collected, 50 μL of which served as Input, and the remaining was incubated with 2 μg antibody to IKZF1 (#5443, 1: 50, Cell Signaling Technologies [CST], Beverly, MA) or IgG (#3423, 1: 20, CST) overnight at 4 °C. Thereafter, the sample was added with 20 μL protein A/G-sepharose microspheres (36403ES03, Yeasen Company, Shanghai, China), and shaken for 3 h at 4 °C. The cells were collected, washed with cell lysate 3 times, heated in 40 μL loading buffer and precipitated for 5 min. Western blot analysis was implemented to quantify the protein expression of HDAC1 (ab53091, 1: 1000, Abcam) and IKZF1 (#5443, 1: 50, CST). The interaction between HDAC1 and IKZF1 proteins in RAW 264.7 cells was detected with the same procedures and same antibodies as the above. The cells were grouped into control, LPS, LPS + PU, LPS + oe-NC, and LPS + oe-HDAC1 groups.

### ChIP

ChIP assay was implemented using RAW264.7 cells through a ChIP Assay Kit (EMD Millipore,). The resulting solutions were incubated with antibodies (HDAC1 (ab7028, 1: 50, Abcam) or IgG (ab171870, 1 μg/mL, Abcam). Finally, the obtained immunoprecipitated DNA was analyzed by RT-qPCR. Primers for PP2A promoter region (−2000 bp - 0 bp): Forward: 5′-GTCAGCTCTTGCCTTGACCT-3′, Reverse: 5′-GCTTAGGGGACAAAGGGGTC-3′.

### Statistical analysis

All data were processed using SPSS 21.0 statistical software (IBM Corp., Armonk, NY). The measurement data were displayed in mean ± standard deviation. All data were evaluated regarding normal distribution using Shapiro-Wilk test while homogeneity of variance was assayed by Levene test. In comparison between two groups, unpaired samples t-test was used for data with homogeneity of variance in normal distribution, Welch’s analysis was adopted for heterogeneity of variance, and Mann Whitney test was for data with skewed distribution. Time-based measurements were performed utilizing two-factor analysis of variance, followed by Bonferroni’s post hoc tests. *p* < 0.05 manifested statistically significant.

## Supplementary information

similarity report

Supplemental Material

Original Data File

## Data Availability

The datasets generated and/or analyzed during our work are available in the manuscript and supplementary materials.

## References

[1] Butt Y, Kurdowska A, Allen TC (2016). Acute lung injury: a clinical and molecular review. Arch Pathol Lab Med.

[2] Herridge MS, Tansey CM, Matte A, Tomlinson G, Diaz-Granados N, Cooper A (2011). Functional disability 5 years after acute respiratory distress syndrome. N. Engl J Med.

[3] Guo H, Xie M, Zhou C, Zheng M (2019). The relevance of pyroptosis in the pathogenesis of liver diseases. Life Sci.

[4] Xu YJ, Zheng L, Hu YW, Wang Q (2018). Pyroptosis and its relationship to atherosclerosis. Clin Chim Acta.

[5] Li D, Ren W, Jiang Z, Zhu L (2018). Regulation of the NLRP3 inflammasome and macrophage pyroptosis by the p38 MAPK signaling pathway in a mouse model of acute lung injury. Mol Med Rep..

[6] Cheng KT, Xiong S, Ye Z, Hong Z, Di A, Tsang KM (2017). Caspase-11-mediated endothelial pyroptosis underlies endotoxemia-induced lung injury. J Clin Invest.

[7] Chauhan D, Vande Walle L, Lamkanfi M (2020). Therapeutic modulation of inflammasome pathways. Immunol Rev.

[8] Mangan MSJ, Olhava EJ, Roush WR, Seidel HM, Glick GD, Latz E (2018). Targeting the NLRP3 inflammasome in inflammatory diseases. Nat Rev Drug Disco.

[9] Ning L, Wei W, Wenyang J, Rui X, Qing G (2020). Cytosolic DNA-STING-NLRP3 axis is involved in murine acute lung injury induced by lipopolysaccharide. Clin Transl Med.

[10] Ying Y, Mao Y, Yao M (2019). NLRP3 inflammasome activation by MicroRNA-495 promoter methylation may contribute to the progression of acute lung injury. Mol Ther Nucleic Acids.

[11] Zhang L, Lu L, Zhong X, Yue Y, Hong Y, Li Y (2019). Metformin reduced NLRP3 inflammasome activity in Ox-LDL stimulated macrophages through adenosine monophosphate activated protein kinase and protein phosphatase 2A. Eur J Pharm.

[12] McHugh WM, Russell WW, Fleszar AJ, Rodenhouse PE, Rietberg SP, Sun L (2016). Protein phosphatase 2A activation attenuates inflammation in murine models of acute lung injury. Am J Physiol Lung Cell Mol Physiol.

[13] Nagpal K, Watanabe KS, Tsao BP, Tsokos GC (2014). Transcription factor Ikaros represses protein phosphatase 2A (PP2A) expression through an intronic binding site. J Biol Chem.

[14] Cao LL, Song X, Pei L, Liu L, Wang H, Jia M (2017). Histone deacetylase HDAC1 expression correlates with the progression and prognosis of lung cancer: a meta-analysis. Med (Baltim).

[15] Li F, Ding J, Cong Y, Liu B, Miao J, Wu D (2020). Trichostatin A alleviated ovarian tissue damage caused by cigarette smoke exposure. Reprod Toxicol.

[16] Chen R, Xue J, Xie M (2012). Puerarin prevents isoprenaline-induced myocardial fibrosis in mice by reduction of myocardial TGF-beta1 expression. J Nutr Biochem.

[17] Wong KH, Li GQ, Li KM, Razmovski-Naumovski V, Chan K (2011). Kudzu root: traditional uses and potential medicinal benefits in diabetes and cardiovascular diseases. J Ethnopharmacol.

[18] Zhou YX, Zhang H, Peng C (2014). Puerarin: a review of pharmacological effects. Phytother Res.

[19] Wang X, Yan J, Xu X, Duan C, Xie Z, Su Z (2018). Puerarin prevents LPS-induced acute lung injury via inhibiting inflammatory response. Micro Pathog.

[20] Guo CJ, Xie JJ, Hong RH, Pan HS, Zhang FG, Liang YM (2019). Puerarin alleviates streptozotocin (STZ)-induced osteoporosis in rats through suppressing inflammation and apoptosis via HDAC1/HDAC3 signaling. Biomed Pharmacother.

[21] Wang K, Zhu X, Zhang K, Yao Y, Zhuang M, Tan C (2017). Puerarin inhibits amyloid beta-induced NLRP3 inflammasome activation in retinal pigment epithelial cells via suppressing ROS-dependent oxidative and endoplasmic reticulum stresses. Exp Cell Res.

[22] Zhang Q, Wu D, Yang Y, Liu T, Liu H (2017). Dexmedetomidine alleviates hyperoxia-induced acute lung injury via inhibiting NLRP3 inflammasome activation. Cell Physiol Biochem.

[23] Kovarova M, Hesker PR, Jania L, Nguyen M, Snouwaert JN, Xiang Z (2012). NLRP1-dependent pyroptosis leads to acute lung injury and morbidity in mice. J Immunol.

[24] Raghawan AK, Ramaswamy R, Radha V, Swarup G (2019). HSC70 regulates cold-induced caspase-1 hyperactivation by an autoinflammation-causing mutant of cytoplasmic immune receptor NLRC4. Proc Natl Acad Sci USA.

[25] Blackwell, DL, Lucas, JW & Clarke, TC. Summary health statistics for U.S. adults: national health interview survey, 2012. Vital Health Stat. 2014;10:1–161. 24819891

[26] Hosseinian N, Cho Y, Lockey RF, Kolliputi N (2015). The role of the NLRP3 inflammasome in pulmonary diseases. Ther Adv Respir Dis.

[27] Ding Z, Zhong R, Xia T, Yang Y, Xing N, Wang W (2020). Advances in research into the mechanisms of Chinese Materia Medica against acute lung injury. Biomed Pharmacother.

[28] Serafini M, Peluso I, Raguzzini A (2010). Flavonoids as anti-inflammatory agents. Proc Nutr Soc.

[29] Zhang L (2019). Pharmacokinetics and drug delivery systems for puerarin, a bioactive flavone from traditional Chinese medicine. Drug Deliv.

[30] Wang C, Yan M, Jiang H, Wang Q, Guan X, Chen J (2016). Protective effects of puerarin on acute lung and cerebrum injury induced by hypobaric hypoxia via the regulation of aquaporin (AQP) via NF-kappaB signaling pathway. Int Immunopharmacol.

[31] Kiely M, Kiely PA (2015). PP2A: the wolf in sheep’s clothing?. Cancers (Basel).

[32] Ferrari E, Bruhn C, Peretti M, Cassani C, Carotenuto WV, Elgendy M (2017). PP2A controls genome integrity by integrating nutrient-sensing and metabolic pathways with the DNA damage response. Mol Cell.

[33] Wlodarchak N, Xing Y (2016). PP2A as a master regulator of the cell cycle. Crit Rev Biochem Mol Biol.

[34] Seshacharyulu P, Pandey P, Datta K, Batra SK (2013). Phosphatase: PP2A structural importance, regulation and its aberrant expression in cancer. Cancer Lett.

[35] Perrotti D, Neviani P (2013). Protein phosphatase 2A: a target for anticancer therapy. Lancet Oncol.

[36] Zhang Y, Zhu P, Wu X, Yuan T, Su Z, Chen S (2021). Microcystin-LR induces NLRP3 inflammasome activation via FOXO1 phosphorylation, resulting in interleukin-1beta secretion and pyroptosis in hepatocytes. Toxicol Sci.

[37] Tang T, Gong T, Jiang W, Zhou R (2018). GPCRs in NLRP3 inflammasome activation, regulation, and therapeutics. Trends Pharm Sci.

[38] Chen J, Wang S, Fu R, Zhou M, Zhang T, Pan W (2018). RIP3 dependent NLRP3 inflammasome activation is implicated in acute lung injury in mice. J Transl Med.

[39] Jia X, Cao B, An Y, Zhang X, Wang C (2019). Rapamycin ameliorates lipopolysaccharide-induced acute lung injury by inhibiting IL-1beta and IL-18 production. Int Immunopharmacol.

[40] Zhang Y, Wang X, Liu Z, Yu L (2018). Dexmedetomidine attenuates lipopolysaccharide induced acute lung injury by targeting NLRP3 via miR-381. J Biochem Mol Toxicol.

[41] Guey B, Bodnar M, Manie SN, Tardivel A, Petrilli V (2014). Caspase-1 autoproteolysis is differentially required for NLRP1b and NLRP3 inflammasome function. Proc Natl Acad Sci USA.

[42] Elliott JM, Rouge L, Wiesmann C, Scheer JM (2009). Crystal structure of procaspase-1 zymogen domain reveals insight into inflammatory caspase autoactivation. J Biol Chem.

[43] Walker NP, Talanian RV, Brady KD, Dang LC, Bump NJ, Ferenz CR (1994). Crystal structure of the cysteine protease interleukin-1 beta-converting enzyme: a (p20/p10)2 homodimer. Cell.

[44] Martinon F, Burns K, Tschopp J (2002). The inflammasome: a molecular platform triggering activation of inflammatory caspases and processing of proIL-beta. Mol Cell.

[45] Ma X, Yan L, Zhu Q, Shao F (2017). Puerarin attenuates cisplatin-induced rat nephrotoxicity: the involvement of TLR4/NF-kappaB signaling pathway. PLoS ONE.

[46] Chueh FS, Chang CP, Chio CC, Lin MT (2004). Puerarin acts through brain serotonergic mechanisms to induce thermal effects. J Pharm Sci.

[47] Zhang Y, Li X, Grailer JJ, Wang N, Wang M, Yao J (2016). Melatonin alleviates acute lung injury through inhibiting the NLRP3 inflammasome. J Pineal Res.

[48] He X, Qian Y, Li Z, Fan EK, Li Y, Wu L (2016). TLR4-upregulated IL-1β and IL-1RI promote alveolar macrophage pyroptosis and lung inflammation through an autocrine mechanism. Sci Rep..

