# Supplementary material for: Puerarin ameliorates acute lung injury by modulating NLRP3 inflammasome-induced pyroptosis
Source: Cell Death Discov. 2022 Aug 18;8:368. doi: 10.1038/s41420-022-01137-8 (PMC9385627; doi:10.1038/s41420-022-01137-8)
Supplement: Supplementary file 1 — similarity report [file 41420_2022_1137_MOESM1_ESM.pdf]

Revised Manuscript .docx

WORD COUNT 5114
TIME SUBMITTED 06-JUL-2022 02:25PM
PAPER ID 88074241

Puerarin ameliorates acute lung injury by modulating NLRP3 inflammasome-induced pyroptosis

Running title: Inhibitory role of PU in ALI

Abstract

We commenced to analyze putative anti-pyroptosis effects of puerarin (PU) as mediated by the PP2A-HDAC1-NLRP3 pathway in acute lung injury (ALI). ALI animal and cell models were constructed, followed by treatment of PU. Then, the effect of HDAC1, PP2A, and NLRP3 on cell inflammation and pyroptosis was explored. The interaction between HDAC1 and PP2A as well as between PP2A and NLRP3 was analyzed. Our findings suggested that PU downregulated HDAC1 expression to alleviate symptoms of ALI. HDAC1 overexpression promoted inflammation induced by LPS, which reversed the inhibitory effect of PU on ALI. HDAC1 overexpression also decreased PP2A expression, suggesting that PP2A was involved in the effects of HDAC1 on LPS-induced inflammation. PP2A exerted inhibitory effects on NLRP3. Meanwhile, PU hindered the progression of ALI by silencing HDAC1 or overexpressing PP2A both *in vivo* and *in vitro*. Taken together, PU restrained pyroptosis of cells induced by NLRP3 inflammasome to abate ALI.

Keywords

Acute lung injury, Cell pyroptosis, Puerarin, HDAC1, PP2A, NLRP3, Inflammasome

INTRODUCTION

Acute lung injury (ALI) refers to a series of lung lesions resulting from multiple lung injuries, which may induce a severe lung condition, known as acute respiratory distress syndrome (ARDS), eventually leading to significant morbidity and death [1]. In addition, a significant proportion of patients who survive ALI/ARDS continue to suffer from physical, cognitive,

and psychological dysfunction [2]. Thus, specific and efficient treatments and drugs for ALI warrant research. Pyroptosis refers to a pro-inflammatory process of cell death regulation that relies on the activity of inflammatory proteases, which are family members of cysteine-dependent aspartate-specific proteases [3]. The primary feature of pyroptosis is a rapidly ruptured cytoplasmic membrane leading to generation of intracellular contents and pro-inflammatory factors [4]. Furthermore, pyroptosis is detected in the ALI/ARDS mouse model and the LPS-induced macrophages [5].

Moreover, the inflammatory caspases may contribute to the pyroptosis [6]. NLRP3 is the key representative of inflammasome, the activation of which may induce Caspase 1-mediated proteolytic activation of the IL-18 and IL-1β, and trigger pyroptosis [7, 8]. As previously documented, NLRP3 inflammasome activation by the sensory receptor of cytosolic DNA is one of the crucial reasons causing lipopolysaccharide (LPS)-induced ALI [9]. Notably, attenuation of activation of the NLRP3 inflammasome is capable of preventing the progression of ALI [10]. Reduction of NLRP3 inflammasome activity in oxidized low-density lipoprotein-stimulated macrophages is associated with the upregulation of protein phosphatase 2A (PP2A) [11]. PP2A activation can prevent inflammation and tissue injury in murine models of ALI [12]. Additionally, IKZF1 can modulate PP2A expression through recruitment of histone deacetylase 1 (HDAC1) [13]. Furthermore, HDAC1 is an essential epigenetic regulator and its inhibitor possesses suppressive role in the activation of pyroptosis [14, 15]. Therefore, we are curious about whether the HDAC1/IKZF1/PP2A axis is involved in regulating NLRP3 inflammasome-induced pyroptosis in ALI.

Pueraria lobata (Willd.) Ohwi is a traditional Chinese medicine, which is also called as Kudzu root [16]. It mainly possesses effects on fever, emesis, diarrhea, liver injury, cardiac dysfunctions, toxicosis, as well as weight loss [17]. Puerarin (PU) is the dominating ingredient with bioactivity extracted from the root of Gegen [18]. A recent study has also reported that PU can inhibit ALI through activating LXRα, which subsequently decreases

LPS-mediated inflammatory response [19]. PU has been demonstrated to serve as an appealing therapeutic method to restrain diabetic osteoporosis due to its inhibiting effect on the HDAC1/HDAC3 signaling [20]. Importantly, PU can inhibit Aβ-induced NLRP3 inflammasome in retinal pigment epithelial cells [21]. Whether PU regulates NLRP3 inflammasome-induced pyroptosis in ALI through the HDAC1/IKZF1/PP2A axis is an interesting question.

Given the aforementioned evidence, this study intended to explore the potential roles of PU in mediating the process of pyroptosis involving HDAC1/PP2A, which eventually may attenuate the inflammatory in ALI and how PU regulates NLRP3 inflammasome-induced pyroptosis in ALI. This research may provide a therapeutic strategy to reduce inflammatory responses and lung injury in ALI.

RESULTS

PU exerted inhibitory effect on ALI and HDAC1 was downregulated in the lung tissues of ALI mice

As previously documented, PU has an inhibitory effect on ALI [19]. A mouse model of ALI was developed to assay the detailed molecular mechanism underlying the inhibitory effect of PU on ALI. As illustrated in Figure 1A, ALI mouse showed significantly thickened alveolar septum, severely damaged lung tissues, and a large number of inflammatory cells infiltrated in the lung tissues, while ALI mice treated with PU showed a slightly widened alveolar septum, basically intact alveolar structure and reduced degree of inflammatory cell infiltration. In addition, the total number of cells and neutrophils as well as the levels of TNF-α, IL-1β, INF-γ, and IL-6 in bronchoalveolar lavage fluid (BALF) were increased in the ALI mice, while PU treatment led to the opposite trends (Figure 1B, C).

Western blot analysis results (Figure 1D, Supplementary Figure 1A) depicted that the expression of HDAC1 in the lung tissues of mice in the ALI group was elevated while

additional PU treatment reduced HDAC1 expression.

Hence, data above indicated that PU presented an inhibitory effect on ALI and HDAC1 was poorly expressed in the lung tissues of ALI mice.

PU inhibited LPS-induced inflammation through HDAC1

We then probed into the mechanism of inhibitory effect of PU on ALI, mouse macrophages RAW 264.7 were used to develop a cell model of inflammation by means of LPS. As displayed by Figure 2A-C and Supplementary Figure 1C, the expression of HDAC1 and the contents of TNF-α, IL-1β, INF-γ and IL-6 in the supernatant were increased in cells treated with LPS, all of which were reversed by further PU treatment.

Then, RAW 264.7 cells were treated with overexpressed HDAC1 (oe-HDAC1), followed by LPS or PU treatment. According to ELISA (Figure 2D, Supplementary Figure 1C), oe-HDAC1 significantly promoted LPS-induced inflammation as evidenced by upregulation of TNF-α, IL-1β, INF-γ and IL-6. Addition of PU alleviated inflammation induced by LPS, which was counterweighed by overexpressing HDAC1.

In summary, PU was involved in the suppression of ALI by reducing HDAC1 expression.

HDAC1 promoted LPS-induced cell inflammation by inhibiting PP2A

For assessing the role of HDAC1 in ALI was related to the expression of PP2A, animal and cell models were constructed. Western blot analysis indicated that the expression of PP2A was diminished in the ALI model but increased after further treatment of PU (Figure 3A, Supplementary Figure 1B). Moreover, in LPS-treated cells, elevated HDAC1 but reduced PP2A levels were observed, and further treatment with PU led to the opposite trends; in LPS-induced cells with oe-HDAC1 treatment, elevated HDAC1 but reduced PP2A were found relative to LPS + negative control for gene overexpression (oe-NC) treatment (Figure

3B). Collectively, the expression of PP2A was reduced in ALI, while PU treatment promoted the expression of PP2A and overexpression of HDAC1 inhibited the expression of PP2A.

In order to confirm the mechanism of HDAC1 modulating PP2A expression, the interaction between HDAC1 and IKZF1 was tested by co-immunoprecipitation (Co-IP) experiments. Data presented obvious interaction between HDAC1 and IKZF1 in 293T cells *in vitro*, and a strong interaction between the two was detected following concomitant overexpression of HDAC1 and IKZF1. In RAW 264.7 cells *in vivo*, interaction between HDAC1 and IKZF1 was also detected and the intensity of this interaction changed with the expression of HDAC1 protein. Specifically, PU treatment profoundly inhibited the protein expression of HDAC1 and the interaction between HDAC1 and IKZF1 was attenuated. However, overexpression of HDAC1 enhanced the interaction between HDAC1 and IKZF1 (Figure 3C, and D). Moreover, the results of chromatin immunoprecipitation (ChIP) assay verified that HDAC1 bound to the promoter region of PP2A (Figure 3E). Furthermore, in cells transduced with oe-HDAC1, an increase in HDAC1 but a reduction in PP2A expression was found (Figure 3F).

Additionally, whether the role of HDAC1 in LPS-induced inflammatory response was related to PP2A was explored with PP2A overexpressed in RAW 264.7 cells using lentivirus. RT-qPCR (Figure 3G) depicted that the expression of PP2A in cells with oe-PP2A treatment was increased. In addition, ELISA results (Figure 3H) noted that the contents of TNF-α, IL-1β, INF-γ and IL-6 were reduced in LPS-induced cell with oe-PP2A treatment alone while HDAC1 overexpression alone elevated the contents of TNF-α, IL-1β, INF-γ and IL-6. Moreover, PP2A overexpression significantly suppressed the contribution of HDAC1 overexpression on cell inflammation.

Collectively, HDAC1 promoted LPS-induced cellular inflammation by inhibiting PP2A.

PP2A inhibited LPS-induced cell inflammation and pyroptosis by reducing NLRP3

inflammasome activation

Previous studies have documented that PP2A inhibits the NLRP3 inflammasome activation [12] and NLRP3 inflammasome promotes ALI [22]. Thus, the expression of NLRP3 inflammasome-related indicators in animal and cell models was first detected by Western blot analysis and the outcomes (Figure 4A, B) indicated that ALI mouse lung tissues and LPS-induced cells showed elevated expression levels of NLRP3, pro-IL-1β, and Caspase-1 p20 protein, but Caspase-1 levels did not significantly change.

To discern that LPS-induced NLRP3 inflammasome activation was related to PP2A, RAW 264.7 cells were transfected with overexpressed PP2A. Western blot analysis was used to detect PP2A and NLRP3 inflammasome-related indicators and results revealed that (Figure 4C) the expression of PP2A in LPS-treated cells upon PP2A treatment was increased, and the expression of NLRP3, pro-IL-1β, and Caspase-1 p20 protein was reduced, while Caspase-1 had no significant change. ELISA identified reduced levels of TNF-α, IL-1β, INF-γ, and IL-6 in LPS-treated cells upon PP2A treatment (Figure 4D).

NLRP1-dependent apoptosis is capable of inducing ALI and morbidity in mice [23]. The formation of apoptosis-associated speck-like protein containing CARD (ASC) pyrosomes is a unique feature of caspase-1 induced pyrosomes [24]. Therefore, we detected the effect of PP2A on pyroptosis. RAW 264.7 cells were treated with LPS and ATP *in vitro* for 0 h, 8 h, 16 h, and 24 h followed by visualization of ASC by florescence-tagged ASC antibody and confocal microscopy. We found that the number of ASC foci positive cells was increased in the LPS-induced cells treated with ATP, while further addition of PP2A brought about contrary trends (Figure 4E).

These results concluded that PP2A inhibited LPS-induced cell inflammation and pyroptosis by limiting the activation of NLRP3 inflammasome.

Overexpression of HDAC1 or downregulation of PP2A *in vivo* inhibited the protective

effect of PU in ALI models

For measuring the mechanism of HDAC1 or PP2A on ALI, a mouse model of ALI was constructed and then the ALI model was treated with overexpressed HDAC1 or silenced PP2A, followed by PU treatment. RT-qPCR (Figure 5A and B) revealed that ALI mice and PU-treated ALI mice upon overexpressed HDAC1 treatment had elevated HDAC1 expression but reduced PP2A. However, relative to ALI mice, PU treatment led to decreased HDAC1 but increased PP2A expression. We also found short hairpin RNA (shRNA) against PP2A (sh-PP2A) treatment caused a reduction on PP2A expression in ALI mice.

Next, it was observed that upon HDAC1 overexpression or PP2A silencing in the PU treated ALI mice, the alveolar septum was significantly thickened and the lung tissues were severely damaged, a large number of inflammatory cells were infiltrated, and the total cell number, neutrophil number in BALF were increased (Figure 5C and D). Detection of the PP2A and cell pyroptosis-related indicators by Western blot analysis demonstrated that upon HDAC1 overexpression or PP2A silencing in the PU treated ALI mice, the expression of PP2A was reduced and the expression of NLRP3, pro-IL-1β, and Caspase-1 p20 protein were increased, while the expression of Caspase-1 presented no significant changes (Figure 5E, Supplementary Figure 1D). These results suggested that overexpressed HDAC1 or silenced PP2A promoted cell pyroptosis in the lung tissues of mice.

Further, ELISA results displayed that after overexpression of HDAC1 or silencing of PP2A in the PU-treated ALI mice, elevated levels of TNF-α, IL-1β, INF-γ, and IL-6 were observed (Figure 5F), indicating that overexpressed HDAC1 or silenced PP2A curbed the alleviation of inflammatory responses by PU in the lung tissues of mice.

These results suggested that upregulating HDAC1 or downregulating PP2A expression *in vivo* inhibited the protective effects of PU on ALI.

DISCUSSION

Respiratory diseases and lung injuries are one of the primary causes of death all around the world [25]. An exaggerated inflammatory response is a critical characteristic of these diseases, including ALI [26]. Chinese medicine has long comprised the traditional approach for the treatment of ALI in China and has been found to be efficient [27]. Flavonoids possess a lot of biological functions and shows definite anti-inflammatory effects [28]. PU is a main bioactive flavone from traditional Chinese medicine [29]. Here, we attempted to uncover the putative role of PU in ALI. Our work emphasized that PU increased the expression of PP2A by downregulating HDAC1, leading to the inhibition of inflammation caused by the activation of the NLRP3 inflammasome (Figure 6).

A previous study has demonstrated the inhibitory effects of PU on ALI [19, 30]. To verify the biological activity of PU on ALI, a mouse model with ALI was generated in our work and the anti-ALI effects of PU was verified *in vivo*. As reported, HDAC1 overexpression exacerbates inflammation and inhibiting HDAC1 prevents fructose-elicited inflammation in cells [20], which was in line with our finding that upregulation of HDAC1 in ALI was reduced by the treatment of PU. Moreover, LPS treatment induced HDAC1 expression in RAW 264.7 ALI cell model and overexpression of HDAC1 promoted the secretion of TNF-α, IL-1β, INF-γ and IL-6. Proinflammatory cytokines TNF-α, IL-1β, INF-γ and IL-6 may be potential biomarkers for predicting the morbidity and mortality of ALI [1]. These results suggested a potential role of HDAC1 in ALI and PU treatment for ALI.

Furthermore, the mechanism of HDAC1 involving PU treatment for ALI was studied. Previous literature has shown that by interacting with IKZF1 and binding to the promoter of PP2A, HDAC1 can inhibit the expression of PP2A [13]. It is also reported that PP2A inhibits ALI [12]. Hence, the function of PP2A in PU-mediated ALI treatment was studied. PP2A is a dominating serine/threonine phosphatase and is extensively expressed in eukaryotic cells [31]. PP2A is of great importance to cell processes, such as cell metabolism, cell cycle, cell

survival and cell migration [32-35]. PP2A activation has been confirmed to abolish the inflammation in murine models of ALI [12]. Inhibition of PP2A contributes to NLRP3 inflammasome activation, resulting in IL-1β secretion and pyroptosis in hepatocytes [36]. In addition, metformin treatment can increase the activity of PP2A to alleviate the pro-inflammatory response in oxidized low-density lipoprotein-stimulated macrophages by blocking NLRP3 inflammasome activation through reducing NLRP3 [11]. The expression of PP2A was inhibited in the model of ALI and PP2A inhibits LPS-induced cellular inflammation and pyroptosis by inhibiting NLRP3 inflammatory bodies in this study.

As reported, PP2A inhibits the NLRP3 inflammasome and PP2A downregulation restores NLRP3 in macrophages [11]. The NLRP3 inflammasome has a crucial role in the process of diverse human inflammatory disorders, including atherosclerosis, diabetes, and Alzheimer's disease [37]. The NLRP3 inflammasome was activated after LPS treatment in this study. It is reported NLRP3 inflammasome activation in infiltrating macrophages is observed in LPS induced ALI [38]. IL-1β takes crucial and detrimental functions in the ALI development [39]. Notably, elevation of cleaved Caspase-1 is detected in lung tissues under the LPS challenge [40]. Caspase-1 autoproteolysis has been essential for NLRP3 inflammasome function [41] [42, 43]. Inflammasomes are able to form the molecular conditions that induce the Caspase-1 dimerization and autoproteolysis [44]. Likewise, we found that PU treatment or PP2A overexpression reduced Caspase-1 p20 protein expression by inhibiting the NLRP3 inflammasome in LPS-induced ALI. The formation of ASC coke is a unique feature of Caspase-1 induced pyroptosis [24]. It is known that NLRP1-dependent pyroptosis results in ALI and disease in mice [23]. Conclusively, PP2A inhibited LPS-induced cell inflammation and pyroptosis, which was modulated by the inhibition of NLRP3 inflammasome.

In conclusion, PU demonstrated anti-ALI activity by inhibiting the pyroptosis of cells, which was mediated by the regulation of the PP2A-HDAC1-NLRP3 inflammasome pathway.

This research provided preliminary evidence suggesting a new mechanism for the PU-based treatment of ALI. In further studies, the dose-response relationship of PU effects on ALI should be addressed.

MATERIALS AND METHODS

Ethical statement

The current study was ratified by the Animal Ethics Committee of the First Hospital of China Medical University. The animal protocols were implemented in the light of the Guide for the Care and Use of Laboratory Animals published by the US National Institutes of Health.

Construction of mouse models of ALI

C57BL/6J mice (aged 6 - 8 weeks; weighed 18 - 22 g) were raised under a 12 h light/dark cycle each day (eat and drink freely). The mice were then randomized into the control, ALI and ALI-PU groups (ALI mice were treated with PU) with 10 mice in each group [45, 46]. The mice in the control group received no treatment. The mice in the ALI group were first anesthetized by intraperitoneal injection of 3% sodium pentobarbital, and a 5 mm median neck incision was made to expose the trachea. A microsyringe was used to instill LPS solution (2.5 mg/kg, L2630, Sigma-Aldrich, St. Louis, MO) from the trachea to the lungs within 1 min. The incision was then sterilized and sutured. The mice were then kept warm. As for the mice in the ALI-PU group, they were intraperitoneally injected with PU (30 mg/kg; P5555, Sigma-Aldrich) intraperitoneally 1 h before the LPS injection.

Meantime, to assess the role of HDAC1 and PP2A in ALI, mice were taken out and injected intraperitoneally with lentivirus-based oe-HDAC1 and sh-PP2A (5×10^8 pfu/100 μL) 6 days before ALI-PU treatment. Synthesis of lentivirus-mediated oe-HDAC1 and sh-PP2A was performed by HanBio Technology (Shanghai, China). The mice were divided into the ALI-PU + oe-NC group (ALI mice were treated with PU and LPS after injection of

lentivirus-mediated oe-NC), the ALI-PU + oe-HDAC1 group (ALI mice were treated with PU and LPS treatment after injection of lentivirus overexpressing HDAC1), the ALI-PU + sh-NC group (ALI mice were treated with PU and LPS treatment after injection of shRNA against NC lentivirus), and the ALI-PU + sh-PP2A group (ALI mice were treated with PU and LPS treatment after injection of shRNA against PP2A lentivirus) with 10 mice in each group.

All mice were euthanized 12 h after LPS treatment and the BALF and lung tissues were harvested for subsequent experimentations.

BALF collection

Mice were euthanized. After exposure of the trachea, tracheal intubation was performed. Phosphate buffer saline (PBS)-ethylenediamine tetraacetic acid (1 mL) was flushed into the bilateral bronchoalveoli of mice through tracheal intubation by a syringe (1 mL). BALF was collected after 3 washes and this collection was repeated for 3 times. Then, the collected BALFs were mixed and centrifuged at 3000 rpm and 4°C for 10 min. BALF supernatant and precipitate were stored separately.

Inflammatory cell count

BALF pellet was lysed with erythrocyte lysis (1 mL) and centrifuged at 3000 rpm and 4°C for 10 min with the supernatant discarded. The pellet was resuspended in 1 mL of PBS. After mixing, 10 μL of the mixture was pipetted onto the cell counting plate. Cells were stained by Wright-Gimsa staining and the inflammatory cells were classified and counted under a light microscope [47].

Hematoxylin-eosin (HE) staining

Tissues from the lower right lung of mice were prepared into paraffin-embedded sections which were stained with hematoxylin for 4 min and counterstained with eosin for 2 min. The

sections were observed under an optical microscope (CSW-PH50, Shenzhen Christie Optical Instruments Co., Ltd. Shenzhen, China) with histopathological changes evaluated and graded in a blind manner utilizing an arbitrary grading scale. The resultant pulmonary parameters were assessed, including hemorrhage, alveolar neutrophil infiltration, infiltration and edema of interstitial and perivascular cells, as well as alveolar epithelial necrosis [48].

ELISA

According to the standard procedures on the instructions, concentrations of TNF-α, IL-1β, IL-6 and IFN-γ in the supernatant were measured using ELISA kits (KS10484, KS10929, KS18212, and KS18210; all form Keshun Biotechnology, Shanghai, China).

Cell culture

RAW 264.7 macrophages (CL-0190, Procell Life Science & Technology Co., Ltd., Wuhan, Hubei, China) derived from the mouse blood were cultured with a high-glucose Dulbecco's modified Eagle's medium (DMEM) replenished with 10% fetal bovine serum, 100 U/mL penicillin and 100 mg/mL streptomycin in a 5% CO_2 incubator at 37°C. RAW 264.7 cells were randomly divided into 3 groups and placed in sterile centrifuge tubes, which were labeled as control (routinely cultured without extra treatment), LPS (treated with 0.5 μg/mL LPS) and LPS-PU (treated with 0.5 μg/mL LPS and 20 μM PU) with 5 × 10^5 cells in each group. Then, cells were cultured in a complete medium (5 mL) for another 24 h. For detection of pyrophysis, cells were treated with 0.5 μg/mL LPS for 0 - 24 h and then with 5 mM ATP (A6559, Sigma-Aldrich) for 30 min.

Immunofluorescence

RAW 264.7 cells (1000 cells) were incubated on a cover glass coated with culture medium, and washed thrice with pre-warmed PBS for 10 min each when reaching 95% - 100% confluence. RAW 264.7 cells were then fixed with 4% paraformaldehyde for 20 min,

immersed in PBS (containing 0.1% Triton X-100) for 10 min, blocked in PBS with 0.1% Tween-20 replenished with 3% bovine serum protein for 2 h and incubated with rabbit polyclonal antibody against TMS1/ASC ASC-speck formation; ab227502, 1 : 100, Abcam Inc., Cambridge, UK) at 4°C overnight. After that, the cells were incubated with Alexa-Fluor 555 conjugated donkey anti-rabbit IgG (ab150074, 1 : 1000, Abcam) for 1 h. Subsequently, the nuclei were stained with 4',6-diamidino-2-phenylindole (D9542, MERCK). Following this, the cells were observed under a CellInsight CX7 LZR confocal microscope (CX7 LZR, Thermo Fisher Scientific Inc., Waltham, MA).

Construction of lentivirus

Primers for PCR were designed based on the mouse HDAC1, PP2A and IKZF1 gene sequences. The primer sequences were: HDAC1 (forward: 5'-GAGCAAGATGGCGCAGACTC-3', reverse: 5'-CTGGTCCCTGGGGACGTTAT-3'), PP2A (forward: 5'-ATTACAGAAAGCCGAGTCCCG-3', reverse: 5'-GCGTCAGCATGCAATGAACT-3') and IKZF1 (forward: 5'-CCAGGATCATTCTTGGCCCC-3', reverse: 5'-AATGCTGCCTGCAAATCCAC-3'). HDAC1, PP2A and IKZF1 overexpression vectors were developed employing pLV-EGFP-N lentivirus, namely pLV-HDAC1, pLV-PP2A and pLV-IKZF1. Meanwhile, PP2A was knocked down using pSIH1-H1-copGFP lentivirus vector (shRNA sequence: 5'-CGACGAGTGTTTAAGGAAATA-3'), being pSIHI-PP2A. pLV-HDAC1, pLV-PP2A, pLV-IKZF1, and pSIHI-PP2A were packaged and purified by HanBio Technology (Shanghai).

Cell transduction and grouping

One day before transduction, RAW 264.7 cells were trypsinized, counted, seeded into 6 wells (2×10^5 cells/well) and then cultured in DMEM in an incubator with 5% CO_2 at 37°C.

Upon 30% - 50% confluence, the original medium was renewed and the serum-free medium (1 mL) containing antibacterial agents was added with 2 × 10^6 TU lentivirus and 5 μg Polybrene (Sigma-Aldrich) for transduction in an incubator with 5% CO_2 at 37°C. The second day, the medium containing lentivirus was removed and 2 mL fresh complete was added for overnight culture. The transduction efficiency was detected under a fluorescence microscope and the transduction rate was calculated: transduction rate = the area of blue-stained cells on the cross section/the area of all cells × 100%. After 48 h of transduction, each well was added with 1 μg/mL puromycin to screen out the stably transduced cells.

Subsequently, stably transduced RAW 264.7 cells were treated with oe-NC (transduced with lentivirus empty vector), oe-HDAC1 (transduced with lentivirus-mediated HDAC1 overexpression vector) oe-PP2A (transduced with lentivirus-mediated PP2A overexpression vector), oe-IKZF1 (transduced with lentivirus-mediated IKZF1 overexpression vector), LPS or/and both LPS and PU (LPS-PU), alone or in combination. The sequences for overexpression vectors are summarized in Supplementary Table 1.

RT-qPCR

TRIzol-extracted total RNA was prepared. RT-qPCR was conducted using SYBRGreen fluorescent dye (RR091A, TAKARA, Japan) on an ABI 7500 qPCR instrument (Applied Biosystems, Foster City, CA). Sequences for all primer pairs (Invitrogen) are summarized in Supplementary Table 2. The relative quantification method was used and β-actin was set as the internal reference. The $2^{-\triangle\triangle Ct}$ method was used to calculate the relative transcription level of target genes.

Western blot analysis

Total protein was extracted, electrophoresed and then electroblotted to a polyvinylidene

fluoride membrane which was incubated with primary antibodies against HDAC1 (ab53091, 1 : 1000, Abcam), PP2A (ab32065, 1 : 500, Abcam), NLRP3 (ab263899, 1 : 1000, Abcam), pro-IL-1β (ab234437, 1 : 1000, Abcam), Caspase-1 (ab138483, 1 : 1000, Abcam), Caspase-1 p20 (sc-398715, 1 : 2000, Santa Cruz Biotechnology, CA) and β-actin (ab8226, 1 : 5000, Abcam, internal reference) at 4°C overnight. Horseradish peroxidase-labeled secondary antibody of goat anti-rabbit IgG (ab97051, 1 : 2000, Abcam) was added for another 1-h of culturing with membrane. The membrane was immersed in an enhanced chemiluminescence reaction solution (BM101, Biomiga) for development.

Co-IP

The interaction between HDAC1 and IKZF1 proteins was verified as follows. The 293T cells (CL-0005, Procell, culture conditions were the same as RAW 264.7 cells) were transfected with oe-NC, oe-HDAC1, oe-IKZF1 or co-transfected with oe-HDAC1 and oe-IKZF1 employing Lipofectamine 2000 reagent (12566014, Thermo Fisher Scientific) for 48 h. Next, cells were lysed with 300 μL cell lysate containing protease inhibitor. The supernatant was collected, 50 μL of which served as Input, and the remaining was incubated with 2 μg antibody to IKZF1 (#5443, 1 : 50, Cell Signaling Technologies [CST], Beverly, MA) or IgG (#3423, 1 : 20, CST) overnight at 4°C. Thereafter, the sample was added with 20 μL protein A/G-sepharose microspheres (36403ES03, Yeasen Company, Shanghai, China), and shaken for 3 h at 4°C. The cells were collected, washed with cell lysate 3 times, heated in 40 μL loading buffer and precipitated for 5 min. Western blot analysis was implemented to quantify the protein expression of HDAC1 (ab53091, 1 : 1000, Abcam) and IKZF1 (#5443, 1 : 50, CST). The interaction between HDAC1 and IKZF1 proteins in RAW 264.7 cells was detected with the same procedures and same antibodies as the above. The cells were grouped into control, LPS, LPS + PU, LPS + oe-NC, and LPS + oe-HDAC1 groups.

ChIP

ChIP assay was implemented using RAW264.7 cells through a ChIP Assay Kit (EMD Millipore,). The resulting solutions were incubated with antibodies (HDAC1 (ab7028, 1 : 50, Abcam) or IgG (ab171870, 1 μg/mL, Abcam). Finally, the obtained immunoprecipitated DNA was analyzed by RT-qPCR. Primers for PP2A promoter region (-2000 bp - 0 bp): Forward: 5'-GTCAGCTCTTGCCTTGACCT-3', Reverse: 5'-GCTTAGGGGACAAAGGGGTC-3'.

9

Statistical analysis

All data were processed using SPSS 21.0 statistical software (IBM Corp., Armonk, NY). The measurement data were displayed in mean ± standard deviation. All data were evaluated regarding normal distribution using Shapiro-Wilk test while homogeneity of variance was assayed by Levene test. In comparison between two groups, unpaired samples 47 t-test was used for data with homogeneity of variance in normal distribution, Welch's analysis was adopted for heterogeneity of variance, and Mann Whitney test was for data with skewed distribution. Time-based measurements were performed utilizing two-factor 13 analysis of variance, followed by Bonferroni's post hoc tests. $p < 0.05$ manifested statistically significant.

Ethics approval

The current study was ratified by the Animal Ethics Committee of the First Hospital of China Medical University. Animal protocols were implemented in the light of the Guide for the Care and Use of Laboratory Animals published by the US National Institutes of Health.

Consent for publication

Not applicable.

Availability of data and materials

The datasets generated and/or analysed during our work are available in the manuscript and supplementary materials.

Competing interests

None.

Funding

None.

Authors' contributions

Dasheng Cai conceived and designed research. Yue Zhao conducted experiments and elaborated all results. Fang Yu assayed data and provided figures. Dasheng Cai and Fang Yu made a draft for the study. Dasheng Cai and Yue Zhao edited manuscript. The final version was approved by all authors.

Acknowledgements

Not applicable.

Figure legends

Figure 1. HDAC1 is expressed at a low level in lung tissues of PU-treated ALI mice. A, pathological changes of lung tissues in animal model, detected by HE staining (× 200). B, total number of cells and neutrophils in BALF of animal model. C, TNF-α, IL-1β, INF-γ and IL-6 levels in the BALF of animal model detected by ELISA. D, HDAC1 expression in lung tissues of animal model detected by western blot analysis. * $p < 0.05$. n = 10.

Figure 2. PU exerts inhibitory effect on LPS-induced inflammation by HDAC1. A, HDAC1 protein expression in RAW 264.7 cells. B, the level of inflammatory factors (TNF-α, IL-1β, INF-γ and IL-6) in RAW 264.7 cell supernatant assayed by ELISA. C, the expression of HDAC1 in RAW 264.7 cells after transduction assayed by western blot analysis. D, the level of lung inflammatory factors (TNF-α, IL-1β, INF-γ and IL-6) in the transduced RAW 264.7 cell supernatant. * $p < 0.05$.

Figure 3. HDAC1 promotes LPS-induced cell inflammation through PP2A. A, PP2A protein expression in lung tissues of animal models assayed by western blot analysis (n = 10). B, the expression of HDAC1 and PP2A in RAW 264.7 cell models, checked by western blot analysis. C, the interaction between HDAC1 and IKZF1 in 293T cells, detected by Co-IP. D, the interaction between HDAC1 and IKZF1 in RAW 264.7 cells detected by Co-IP. E, the enrichment of HDAC1 in the PP2A HDAC1 bound to the promoters region, verified by ChIP. F, the effect of HDAC1 on the expression of HDAC1 and PP2A in RAW 264.7 cells assessed by western blot analysis. G, the effect of PP2A overexpression in RAW 264.7 cells checked by RT-qPCR. H, the levels of lung inflammatory factors (TNF-α, IL-1β, INF-γ and IL-6) in RAW 264.7 cell supernatant after transduction evaluated by ELISA. * $p < 0.05$.

Figure 4. The inhibitory effect of overexpressed PP2A on LPS-induced cellular inflammation and pyroptosis. A, the expression of NLRP3 inflammasome-related indicators in lung tissues of animal models (n = 10), detected by western blot analysis. B, the expression of NLRP3 inflammasome-related indicators in cell models, detected by western blot analysis. C, the expression of PP2A and NLRP3 inflammasome-related indicators after overexpression of PP2A, detected by western blot analysis. D, the levels of lung inflammatory factors (TNF-α, IL-1β, INF-γ and IL-6) after overexpression of PP2A, detected by ELISA. E, the formation of pyrosomes, detected by immunofluorescence (× 400). * $p < 0.05$.

Figure 5. Overexpressed HDAC1 or silencing PP2A suppresses the protective effect of PU on ALI in mice. A, the expression of HDAC1 in lung tissues of ALI mice after PU, oe-HDAC1 or sh-PP2A treatment, assayed by RT-qPCR. B, the expression of PP2A in lung tissues of ALI mice after PU, oe-HDAC1 or sh-PP2A treatment, assayed by RT-qPCR. C, the pathological changes in animal models and lung tissues after transfection (× 200), assessed by HE staining. D, the total number of cells and the number of neutrophils in BALF in animal models after transfection. E, the expression of PP2A and NLRP3 inflammasome-related indicators in lung tissues of animal models after transfection, assayed by Western blot analysis. F, the levels of inflammatory factors (TNF-α, IL-1β, INF-γ and IL-6) in BALF of animal models after transfection, assayed by ELISA. * $p < 0.05$. n = 10.

Figure 6 Schematic map of role of Puerarin in ALI. Puerarin ameliorated ALI by modulating NLRP3 inflammasome-induced pyroptosis.

Revised Manuscript .docx

ORIGINALITY REPORT

19%
SIMILARITY INDEX

PRIMARY SOURCES

1	cellandbioscience.biomedcentral.com<br>Internet	105 words — 2%
2	www.researchsquare.com<br>Internet	82 words — 1%
3	Yue Liu, You-Ping Li, Li-Min Xiao, Li-Ke Chen, Su-Yue Zheng, Er-Ming Zeng, Chun-Hua Xu. "Extracellular vesicles derived from M2 microglia reduce ischemic brain injury through microRNA-135a-5p/TXNIP/NLRP3 axis", Laboratory Investigation, 2021<br>Crossref	60 words — 1%
4	downloads.hindawi.com<br>Internet	43 words — 1%
5	tessera.spandidos-publications.com<br>Internet	42 words — 1%
6	Shuyao Zhang, Xinmin Guan, Wei Liu, Zhe Zhu et al. "YTHDF1 alleviates sepsis by upregulating WWP1 to induce NLRP3 ubiquitination and inhibit caspase-1-dependent pyroptosis", Cell Death Discovery, 2022<br>Crossref	41 words — 1%
7	www.jcancer.org<br>Internet	38 words — 1%

8 hdl.handle.net
Internet
37 words — 1%

9 Qiqin Song, Hongyue Zhang, Jinan He, Hongyan Kong et al. "Long non-coding RNA LINC00473 acts as a microRNA-29a-3p sponge to promote hepatocellular carcinoma development by activating Robo1-dependent PI3K/AKT/mTOR signaling pathway", Therapeutic Advances in Medical Oncology, 2020
Crossref
35 words — 1%

10 link.springer.com
Internet
35 words — 1%

11 www.mdpi.com
Internet
34 words — 1%

12 Yongxiang Li, Huiru Zhu, Lingyun Pan, Bo Zhang, Haixia Che. "microRNA-103a-3p confers protection against lipopolysaccharide-induced sepsis and consequent multiple organ dysfunction syndrome by targeting HMGB1", Infection, Genetics and Evolution, 2021
Crossref
33 words — 1%

13 www.aging-us.com
Internet
27 words — < 1%

14 Qingzeng Qian, Xiangke Cao, Bin Wang, Xiaoliu Dong, Jian Pei, Ling Xue, Fumin Feng. "RETRACTED ARTICLE: Endoplasmic reticulum stress potentiates the autophagy of alveolar macrophage to attenuate acute lung injury and airway inflammation", Cell Cycle, 2020
Crossref
26 words — < 1%

15 www.spandidos-publications.com
Internet
26 words — < 1%

16 www.hindawi.com
Internet
25 words — < 1%

17 cyberleninka.org
Internet
22 words — < 1%

18 cancerci.biomedcentral.com
Internet
21 words — < 1%

19 Yuriko Tachida, Junko Iijima, Kazuto Takahashi, Hideaki Suzuki et al. "Non-classical glycosylation determines intracellular trafficking of APP and Aβ production", Cold Spring Harbor Laboratory, 2022
Crossref Posted Content
20 words — < 1%

20 journals.athmsi.org
Internet
18 words — < 1%

21 Mengdi Li, Shuheng Huang, Yong Zhang, Zhi Song, Haijun Fu, Zhengmei Lin, Xin Huang. "Regulation of the unfolded protein response transducer IRE1α by SERPINH1 aggravates periodontitis with diabetes mellitus via prolonged ER stress", Cellular Signalling, 2022
Crossref
17 words — < 1%

22 jeccr.biomedcentral.com
Internet
17 words — < 1%

23 www.e-fas.org
Internet
17 words — < 1%

24 Lei Zhou, Tieqi Zhang, Shiwei Sun, Yueming Yu, Minghai Wang. "Cryptochrome 1 promotes osteogenic differentiation of human osteoblastic cells via Wnt/β-Catenin signaling", Life Sciences, 2018
Crossref
15 words — < 1%

25 www.thno.org
Internet
14 words — < 1%

26 Jun Dai, Xiaoqi Dong, Rui Liu, Biao Chen, Xiyuan Dong, Quan Wang, Jing-Jing Hu, Fan Xia, Xiaoding Lou. "A peptide-AIEgen nanocomposite mediated whole cancer immunity cycle-cascade amplification for improved immunotherapy of tumor", Biomaterials, 2022
Crossref
13 words — < 1%

27 Danbin Wu, Yefei Chen, Yingxin Sun, Qing Gao, Huhu Li, Zhengfei Yang, Yangxue Wang, Xijuan Jiang, Bin Yu. "Target of MCC950 in Inhibition of NLRP3 Inflammasome Activation: a Literature Review", Inflammation, 2019
Crossref
12 words — < 1%

28 cmjournal.biomedcentral.com
Internet
12 words — < 1%

29 docksci.com
Internet
12 words — < 1%

30 Yongxiang Li, Huiru Zhu, Lingyun Pan, Bo Zhang, Haixia Che. "microRNA-103a-3p confers protection against lipopolysaccharide-induced sepsis and consequent multiple organ dysfunction syndrome by targeting HMGB1", Infection, Genetics and Evolution, 2020
Crossref
11 words — < 1%

31 experiments.springernature.com
Internet
11 words — < 1%

32 Min Shen, Shan Wang, Xin Wen, Xin-Rui Han, Yong-Jian Wang, Xiu-Min Zhou, Man-He Zhang, Dong-Mei Wu, Jun Lu, Yuan-Lin Zheng. "Dexmedetomidine
10 words — < 1%

exerts neuroprotective effect via the activation of the PI3K/Akt/mTOR signaling pathway in rats with traumatic brain injury", Biomedicine & Pharmacotherapy, 2017
Crossref

33 Ronghua Liu, Yucan Zhang, Peiwen Sun, Changxiu Wang. "DDP-resistant ovarian cancer cells-derived exosomal microRNA-30a-5p reduces the resistance of ovarian cancer cells to DDP", Open Biology, 2020 — 10 words — < 1%
Crossref

34 Shilei Zhao, Lei Cui, Xiufeng Zheng, Ying Ji, Chengyuan Yu. "Meloxicam Alleviates Sepsis-Induced Kidney Injury by Suppression of Inflammation and Apoptosis via Upregulating GPNMB", Applied Bionics and Biomechanics, 2022 — 10 words — < 1%
Crossref

35 Tiantian Tang, Xueting Lang, Congfei Xu, Xiaqiong Wang et al. "CLICs-dependent chloride efflux is an essential and proximal upstream event for NLRP3 inflammasome activation", Nature Communications, 2017 — 10 words — < 1%
Crossref

36 Yaozhen Chen, Xiangyang Qin, Qunxing An, Jing Yi et al. "Mesenchymal Stromal Cells Directly Promote Inflammation by Canonical NLRP3 and Non-canonical Caspase-11 Inflammasomes", EBioMedicine, 2018 — 10 words — < 1%
Crossref

37 nanoscalereslett.springeropen.com — 10 words — < 1%
Internet

38 www.degruyter.com — 10 words — < 1%
Internet

39 Bao Yuan, Jing Yang, Hong Gu, Chaoqun Ma. "Down-Regulation of LINC00460 Represses Metastasis of Colorectal Cancer via WWC2", Digestive Diseases and Sciences, 2019
Crossref
9 words — < 1%

40 Xiangxi Kong, Yajin Liao, Lujun Zhou, Ying Zhang, Jinbo Cheng, Zengqiang Yuan, Shukun Wang. "Hematopoietic Cell Kinase (HCK) Is Essential for NLRP3 Inflammasome Activation and Lipopolysaccharide-Induced Inflammatory Response In Vivo", Frontiers in Pharmacology, 2020
Crossref
9 words — < 1%

41 srch.eurekalert.org
Internet
9 words — < 1%

42 www.frontiersin.org
Internet
9 words — < 1%

43 Jiamin Miao, Xuelong Zhou, Tianjiao Ji, Gang Chen. "NF-κB p65-dependent transcriptional regulation of histone deacetylase 2 contributes to the chronic constriction injury-induced neuropathic pain via the microRNA-183/TXNIP/NLRP3 axis", Journal of Neuroinflammation, 2020
Crossref
8 words — < 1%

44 academic.oup.com
Internet
8 words — < 1%

45 aging-us.com
Internet
8 words — < 1%

46 assets.researchsquare.com
Internet
8 words — < 1%

livrepository.liverpool.ac.uk

47 Internet

8 words — < 1%

48 mdpi-res.com
Internet

8 words — < 1%

49 oceanrep.geomar.de
Internet

8 words — < 1%

50 www.nature.com
Internet

8 words — < 1%

51 Jihee Lee Kang, Changsuk Moon, Hui Su Lee, Hae Won Lee, Eun-Mi Park, Hee Sun Kim, Vincent Castranova. "Comparison of the Biological Activity Between Ultrafine and Fine Titanium Dioxide Particles in RAW 264.7 Cells Associated with Oxidative Stress", Journal of Toxicology and Environmental Health, Part A, 2008
Crossref

7 words — < 1%

52 Min Du. "VEGF gene expression is regulated post-transcriptionally in macrophages", FEBS Journal, 2/2006
Crossref

7 words — < 1%

53 Haofeng Liu, Yuan Chen, Linsen Zhou, Xiaohui Jiang, Xiaojun Zhou. "MicroRNA-642b-3p functions as an oncomiR in gastric cancer by down-regulating the CUB and sushi multiple domains protein 1/smad axis", Bioengineered, 2022
Crossref

6 words — < 1%

54 Xiao-Yan Luo, Jian-Hua Ying, Qiao-Sheng Wang. "miR-25-3p ameliorates SAE by targeting the TLR4/NLRP3 axis", Metabolic Brain Disease, 2022
Crossref

6 words — < 1%

EXCLUDE QUOTES	OFF	EXCLUDE SOURCES	OFF
EXCLUDE BIBLIOGRAPHY	OFF	EXCLUDE MATCHES	OFF
