# Supplementary material for: Puerarin ameliorates acute lung injury by modulating NLRP3 inflammasome-induced pyroptosis
Source: Cell Death Discov. 2022 Aug 18;8:368. doi: 10.1038/s41420-022-01137-8 (PMC9385627; doi:10.1038/s41420-022-01137-8)
Supplement: Supplementary file 2 — Supplemental Material [file 41420_2022_1137_MOESM2_ESM.docx]

**Supplementary Table 1.** The sequences for overexpression vectors

| **Sequences for overexpression vector of HDAC1** |
| --- |
| GAGCAAGATGGCGCAGACTCAGGGCACCAAGAGGAAAGTCTGTTACTACTACGACGGGGATGTTGGAAACTACTATTATG |
| GACAAGGGCACCCCATGAAGCCTCACCGAATCCGCATGACTCACAATTTGCTGCTCAACTATGGTCTCTACCGAAAAATG |
| GAGATCTACCGTCCTCACAAAGCCAATGCTGAGGAGATGACCAAGTACCACAGTGATGACTACATTAAATTCCTGCGTTC |
| TATTCGCCCAGATAATATGTCTGAATACAGCAAGCAGATGCAGAGATTCAATGTTGGTGAGGACTGTCCGGTGTTTGATG |
| GCTTGTTTGAGTTCTGTCAGTTGTCCACGGGAGGCTCTGTCGCAAGTGCTGTGAAGCTTAATAAGCAGCAGACGGACATC |
| GCTGTGAACTGGGCCGGGGGCCTGCACCATGCAAAGAAGTCTGAAGCTTCTGGCTTCTGTTACGTCAATGACATCGTCCT |
| GGCCATCCTGGAACTGCTAAAGTACCACCAGAGGGTGCTCTATATTGACATTGATATTCACCATGGCGATGGCGTGGAAG |
| AGGCCTTCTATACTACAGACCGGGTCATGACTGTGTCCTTTCATAAATACGGAGAGTACTTCCCAGGAACTGGGGACCTA |
| CGGGACATTGGGGCTGGCAAAGGCAAGTACTATGCTGTGAACTACCCACTGCGAGACGGCATTGACGACGAATCCTATGA |
| AGCCATCTTTAAGCCAGTCATGTCCAAAGTAATGGAGATGTTCCAGCCTAGTGCAGTGGTCTTACAGTGTGGCTCAGATT |
| CCCTGTCTGGGGACCGGTTAGGTTGCTTCAATCTGACCATCAAAGGACACGCCAAGTGTGTGGAGTTCGTGAAGAGTTTC |
| AACTTGCCCATGCTGATGCTGGGAGGAGGTGGCTACACCATCCGGAATGTTGCTCGCTGCTGGACTTACGAAACAGCGGT |
| GGCCCTGGACACAGAGATCCCTAATGAGCTGCCCTACAACGACTACTTTGAATACTTTGGACCGGATTTCAAGCTTCACA |
| TCAGCCCTTCCAACATGACCAACCAGAACACTAACGAGTACCTGGAGAAGATCAAGCAGCGTCTCTTTGAGAACTTGAGG |
| ATGCTGCCCCATGCCCCTGGGGTCCAGATGCAGGCCATCCCTGAGGATGCCATCCCCGAAGAGAGTGGGGATGAAGATGA |
| GGAGGACCCTGACAAACGCATCTCCATCTGCTCCTCTGACAAACGCATTGCCTGTGAGGAAGAGTTCTCGGACTCAGATG |
| AGGAGGGAGAAGGTGGTCGCAAGAACTCTTCTAACTTCAAAAAAGCCAAAAGAGTTAAAACAGAGGATGAGAAAGAGAAA |
| GATCCTGAAGAGAAAAAAGAAGTCACAGAAGAAGAGAAAACCAAGGAGGAGAAGCCAGAAGCCAAAGGGGTCAAAGAAGA |
| GGTCAAGTTGGCCTGAGCAAGGTCTGCAGCCCCATCTTCTCCCCAAGTTCCTCACTTCTCAGATTTTATATTTTCTATTC |
| CTCTGTGTATTTATATAAAATATATTAACTATAACGTCCCCAGGGACCAG |

Note: HDAC1, histone deacetylase 1.

| **Sequences for overexpression vector of PP2A** |
| --- |
| ATTACAGAAAGCCGAGTCCCGAGCTAGGGCGAGCGGAGGAGGAGGCACAGCGGCCGGCGGCCGAGCACTGCGGAGCGAGC |
| CAGCGGGCCGGCGCCAGCGCCCAGCAGCCGCCTGGGGCCGCAGAAAGCACCCCGGGAGAGCGGCGGCGGCGTGTGCGTGT |
| GGCCCGGGTGCGGGCGGCGGCGCGGGAGCAGCGCGGAGCGGCAGCCGGTTCGGGCGGGCGGCATCATGGACGAGAAGTTG |
| TTCACCAAGGAGCTGGACCAGTGGATCGAGCAGCTGAACGAGTGCAAGCAGCTCTCCGAGTCCCAGGTCAAGAGCCTCTG |
| CGAGAAGGCTAAAGAAATCCTGACAAAAGAATCCAACGTTCAAGAGGTTCGATGTCCAGTCACTGTGTGTGGAGATGTAC |
| ATGGGCAATTTCATGATCTCATGGAACTCTTTAGAATTGGTGGTAAATCACCAGATACAAATTACCTGTTTATGGGAGAC |
| TATGTGGACAGAGGATATTACTCTGTTGAAACAGTTACACTGCTTGTAGCTCTTAAGGTTCGTTACCGAGAGCGCATCAC |
| CATACTCCGAGGGAATCACGAGAGCAGACAGATCACACAGGTTTATGGGTTCTACGACGAGTGTTTAAGGAAATACGGAA |
| ATGCAAATGTTTGGAAATACTTCACAGACCTTTTTGACTATCTTCCTCTCACTGCCTTGGTGGATGGGCAGATCTTCTGT |
| CTACACGGTGGTCTGTCACCATCCATAGACACACTGGATCACATCCGAGCACTCGATCGCCTACAGGAAGTTCCTCATGA |
| GGGTCCAATGTGTGACTTGCTGTGGTCAGATCCAGATGACCGTGGTGGCTGGGGGATATCTCCTCGGGGAGCTGGTTATA |
| CCTTTGGCCAAGATATTTCTGAGACATTTAATCATGCCAATGGCCTCACGTTGGTGTCCAGAGCTCACCAGCTGGTGATG |
| GAGGGATATAACTGGTGCCATGACCGGAACGTAGTAACAATTTTCAGTGCTCCAAACTATTGCTATCGTTGTGGTAACCA |
| AGCTGCAATCATGGAACTTGACGACACTCTTAAGTATTCTTTCTTGCAGTTTGACCCAGCACCTCGTAGAGGCGAGCCAC |
| ATGTCACTCGTCGTACCCCAGACTACTTCCTGTAATGAAAATGTAAACTTGTACAGTATTGCCATGAACCGTATATTGAC |
| CTAATGGAAATGGGAAGAGCAACAGTAACTCCAAAGTGTCAGAAAATAGTTAACATTCAAAAACTTGTTTTCACACGGAC |
| CAAAAGATGTGCCATATAAAATACAAAGCCTCTTGTCATCAACAGCCGTGACCACTTTAGAATGAACCAGTTCATTGCAT |
| GCTGACGC |

Note: PP2A, protein phosphatase 2A.

| **Sequences for overexpression vector of IKZF1** |
| --- |
| CCAGGATCATTCTTGGCCCCCAAAGCGCGGCGCACAAATCCACATAACCTGAAGACAATGGATGTCGATGAGGGTCAAGA |
| CATGTCCCAAGTTTCAGGAAAGGAGAGCCCCCCAGTCAGTGACACTCCAGATGAAGGGGATGAGCCCATGCCTGTCCCTG |
| AGGACCTGTCCACTACCTCTGGAGCACAGCAGAACTCCAAGAGTGATCGAGGCATGGCCAGTAATGTTAAAGTAGAGACT |
| CAGAGTGATGAAGAGAATGGGCGTGCCTGTGAAATGAATGGGGAAGAATGTGCAGAGGATTTACGAATGCTTGATGCCTC |
| GGGAGAGAAAATGAATGGCTCCCACAGGGACCAAGGCAGCTCGGCTTTGTCAGGAGTTGGAGGCATTCGACTTCCTAACG |
| GAAAACTAAAGTGTGATATCTGTGGGATCGTTTGCATCGGGCCCAATGTGCTCATGGTTCACAAAAGAAGTCATACTGGT |
| GAACGGCCTTTCCAGTGCAACCAGTGTGGGGCCTCCTTTACCCAGAAAGGCAACCTCCTGCGGCACATCAAGCTGCACTC |
| GGGTGAGAAGCCCTTCAAATGCCATCTTTGCAACTATGCCTGCCGCCGGAGGGACGCCCTCACCGGCCACCTGAGGACGC |
| ACTCCGTTGGTAAGCCTCACAAATGTGGATATTGTGGCCGGAGCTATAAACAGCGAAGCTCTTTAGAGGAGCATAAAGAG |
| CGATGCCACAACTACTTGGAAAGCATGGGCCTTCCGGGCATGTACCCAGTCATTAAGGAAGAAACTAACCACAACGAGAT |
| GGCAGAAGACCTGTGCAAGATAGGAGCAGAGAGGTCCCTTGTCCTGGACAGGCTGGCAAGCAATGTCGCCAAACGTAAGA |
| GCTCTATGCCTCAGAAATTTCTTGGAGACAAGTGCCTGTCAGACATGCCCTATGACAGTGCCAACTATGAGAAGGAGGAT |
| ATGATGACATCCCACGTGATGGACCAGGCCATCAACAATGCCATCAACTACCTGGGGGCTGAGTCCCTGCGCCCATTGGT |
| GCAGACACCCCCCGGTAGCTCCGAGGTGGTGCCAGTCATCAGCTCCATGTACCAGCTGCACAAGCCCCCCTCAGATGGCC |
| CCCCACGGTCCAACCATTCAGCACAGGACGCCGTGGATAACTTGCTGCTGCTGTCCAAGGCCAAGTCTGTGTCATCGGAG |
| CGAGAGGCCTCCCCGAGCAACAGCTGCCAAGACTCCACAGATACAGAGAGCAACGCGGAGGAACAGCGCAGCGGCCTTAT |
| CTACCTAACCAACCACATCAACCCGCATGCACGCAATGGGCTGGCTCTCAAGGAGGAGCAGCGCGCCTACGAGGTGCTGA |
| GGGCGGCCTCAGAGAACTCGCAGGATGCCTTCCGTGTGGTCAGCACGAGTGGCGAGCAGCTGAAGGTGTACAAGTGCGAA |
| CACTGCCGCGTGCTCTTCCTGGATCACGTCATGTATACCATTCACATGGGCTGCCATGGCTTTCGGGATCCCTTTGAGTG |
| TAACATGTGTGGTTATCACAGCCAGGACAGGTACGAGTTCTCATCCCATATCACGCGGGGGGAGCATCGTTACCACCTGA |
| GCTAAACCCAGCCAGGCCCCACTGAAGCACAAAGATAGCTGGTTATGCCTCCTTCCCGGCAGCTGGACCCACAGCGGACA |
| ATGTTGGGAGTGGATTTGCAGGCAGCATT |

Note: IKZF1, IKAROS family zinc finger 1.

**Supplementary Table 2** Primer sequences for RT-qPCR

| Target gene | Primer sequence |
| --- | --- |
| HDAC1 | F: 5’-ATTCCTGCGTTCTATTCGCCCAGA-3’ |
|  | R: 5’-TTAGCAGTTCCAGGATGGCCAAGA-3’ |
| PP2A | F: 5’-ATGGACGAGAAGTTGTTCACC-3’ |
|  | R: 5’-CAGTGACTGGACATCGAACCT-3’ |
| β-actin | F: 5’-GAGAAGATCTGGCACCACACC-3’ |
|  | R: 5’-GCATACAGGGACAGCACAGC-3’ |

Notes: RT-qPCR, reverse transcription quantitative polymerase chain reaction; HDAC1, histone deacetylase 1; PP2A: protein phosphatase 2A; F, forward; R, reverse.

**
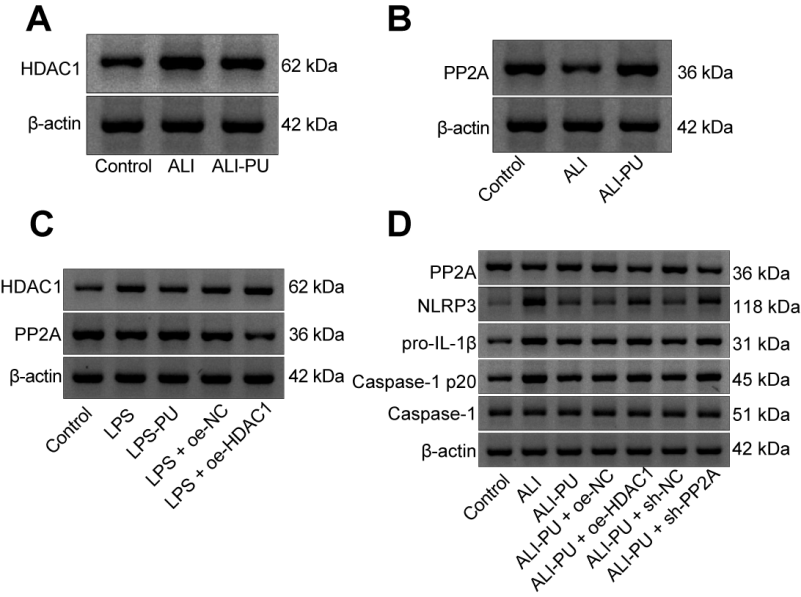
**

**Supplementary Figure 1.** A, Representative protein bands of Figure 1D. B, Representative protein bands of Figure 3A. C, Representative protein bands of Figure 2C. D, Representative protein bands of Figure 5E.
