# Supplementary material for: Puerarin ameliorates acute lung injury by modulating NLRP3 inflammasome-induced pyroptosis
Source: Cell Death Discov. 2022 Aug 18;8:368. doi: 10.1038/s41420-022-01137-8 (PMC9385627; doi:10.1038/s41420-022-01137-8)
Supplement: Supplementary file 3 — Original Data File [file 41420_2022_1137_MOESM3_ESM.docx]

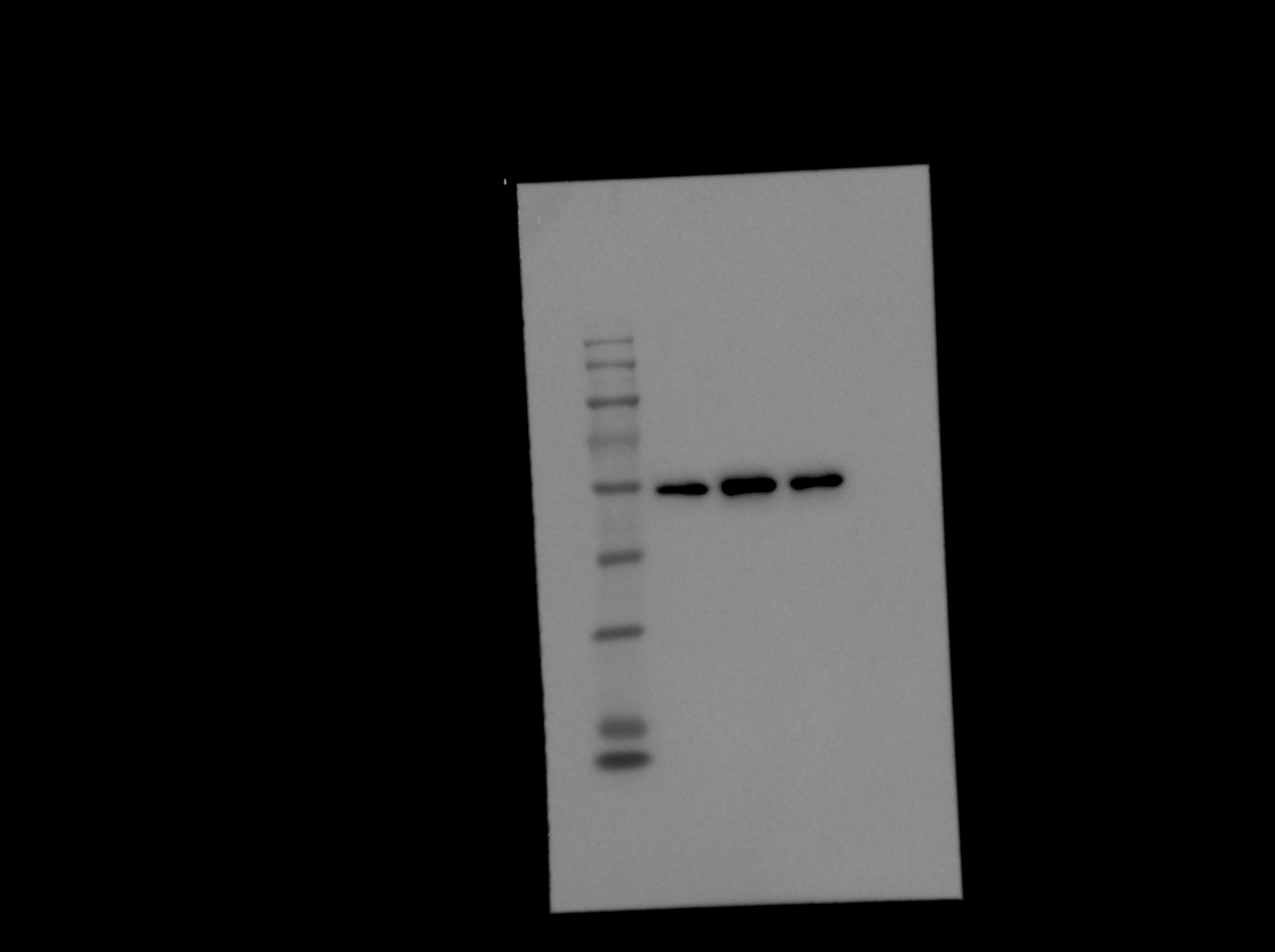


Figure1D


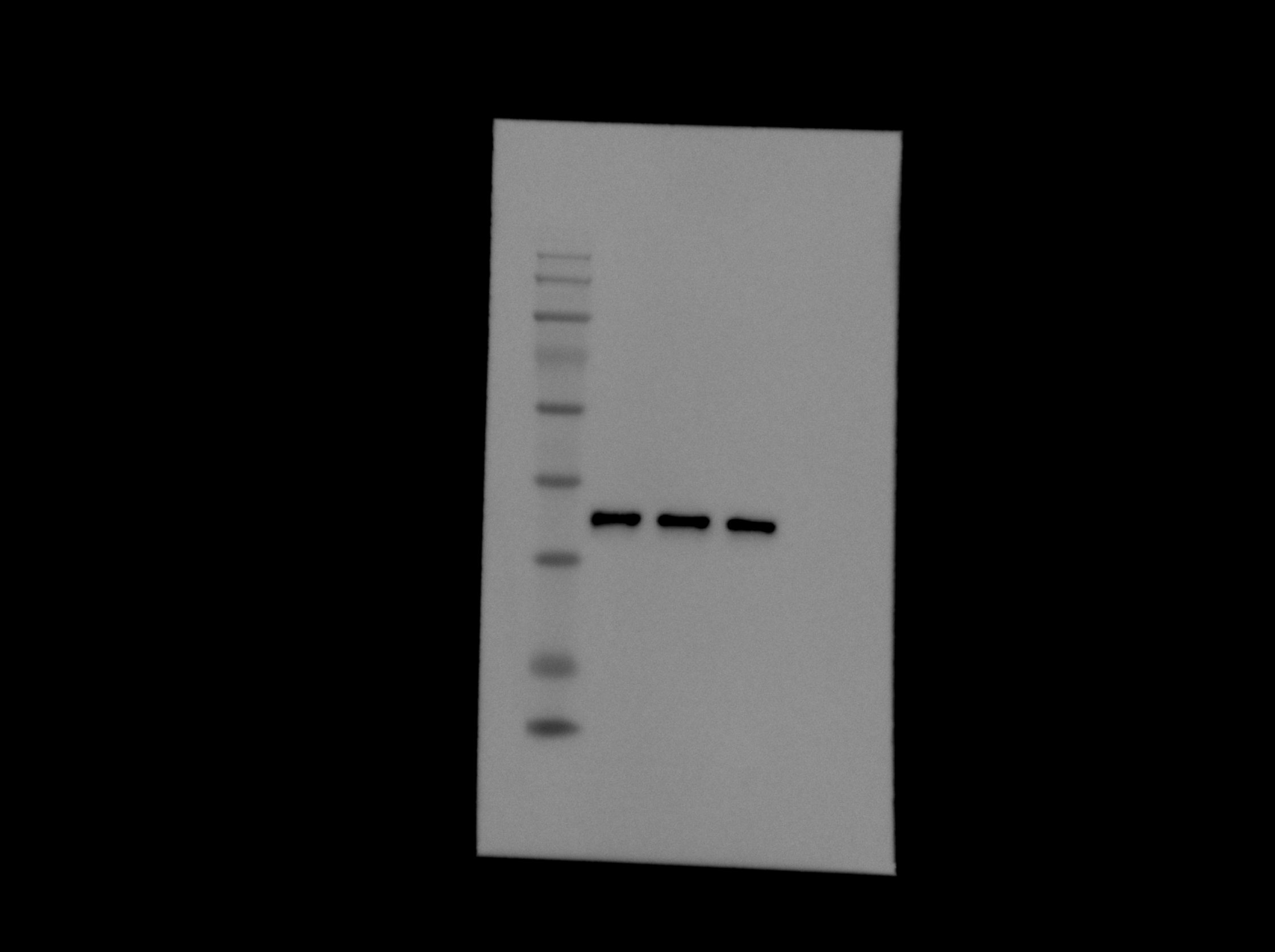


Figure1D-2


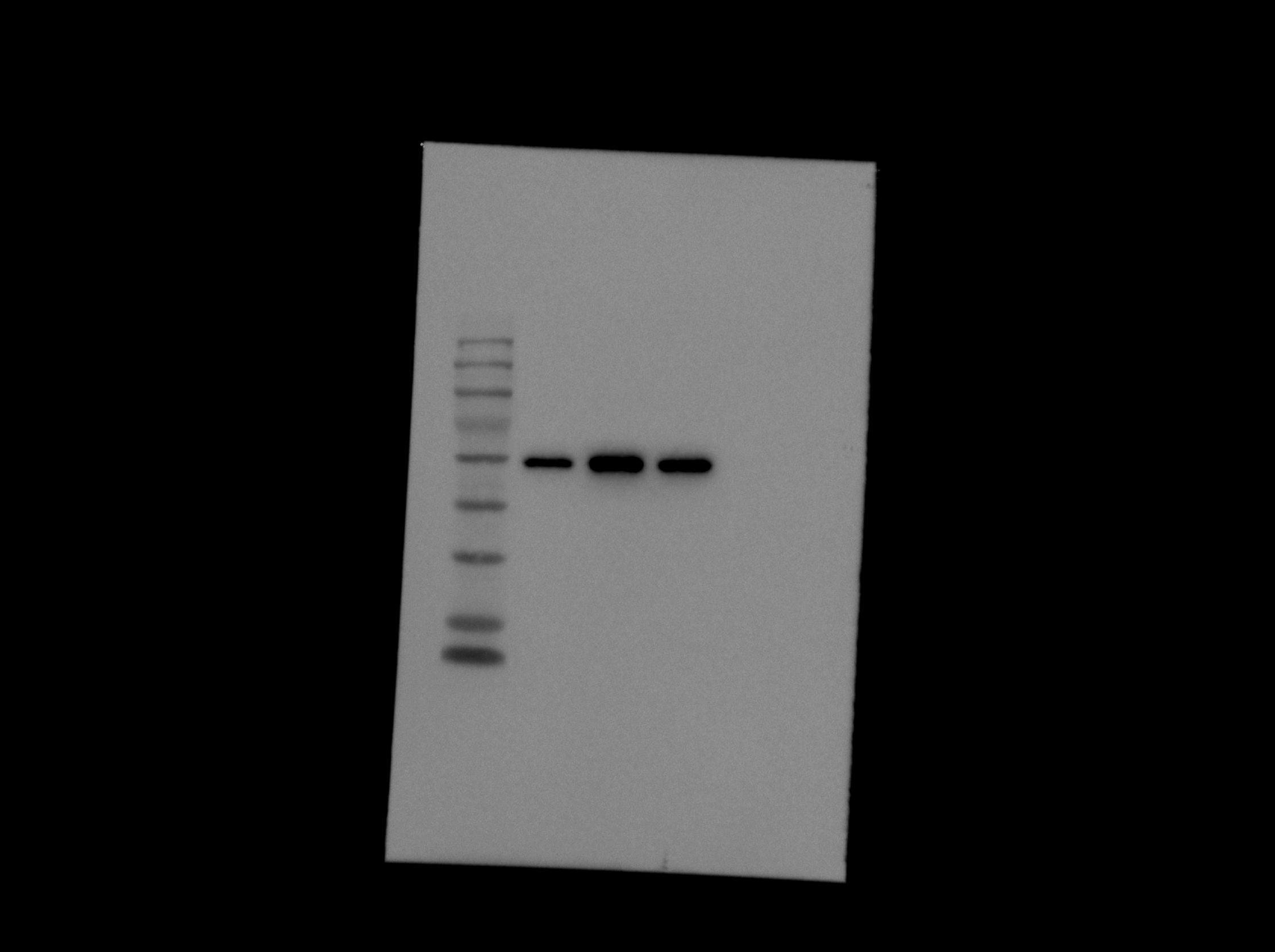


Figure2A-1


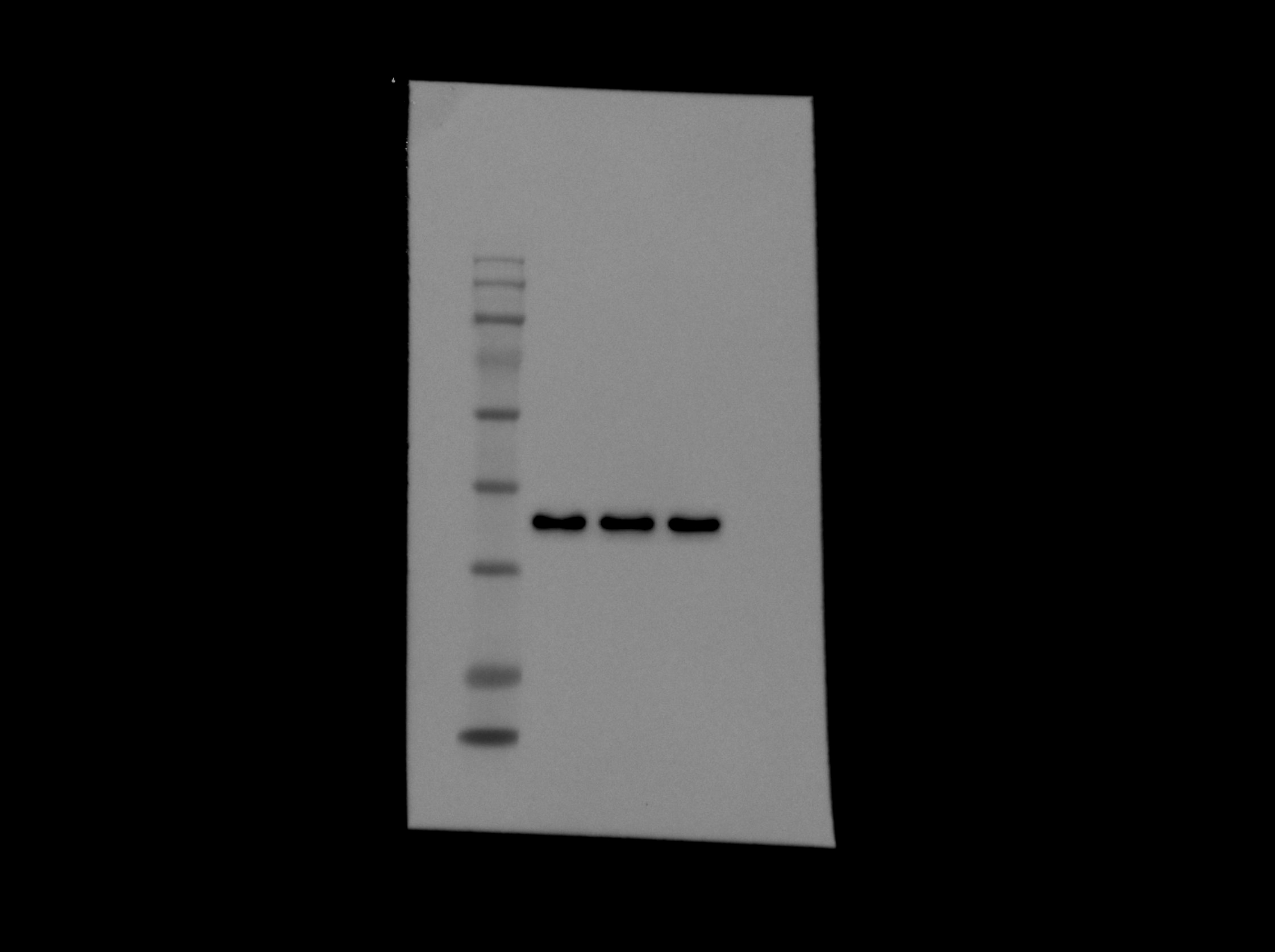


Figure2A-2


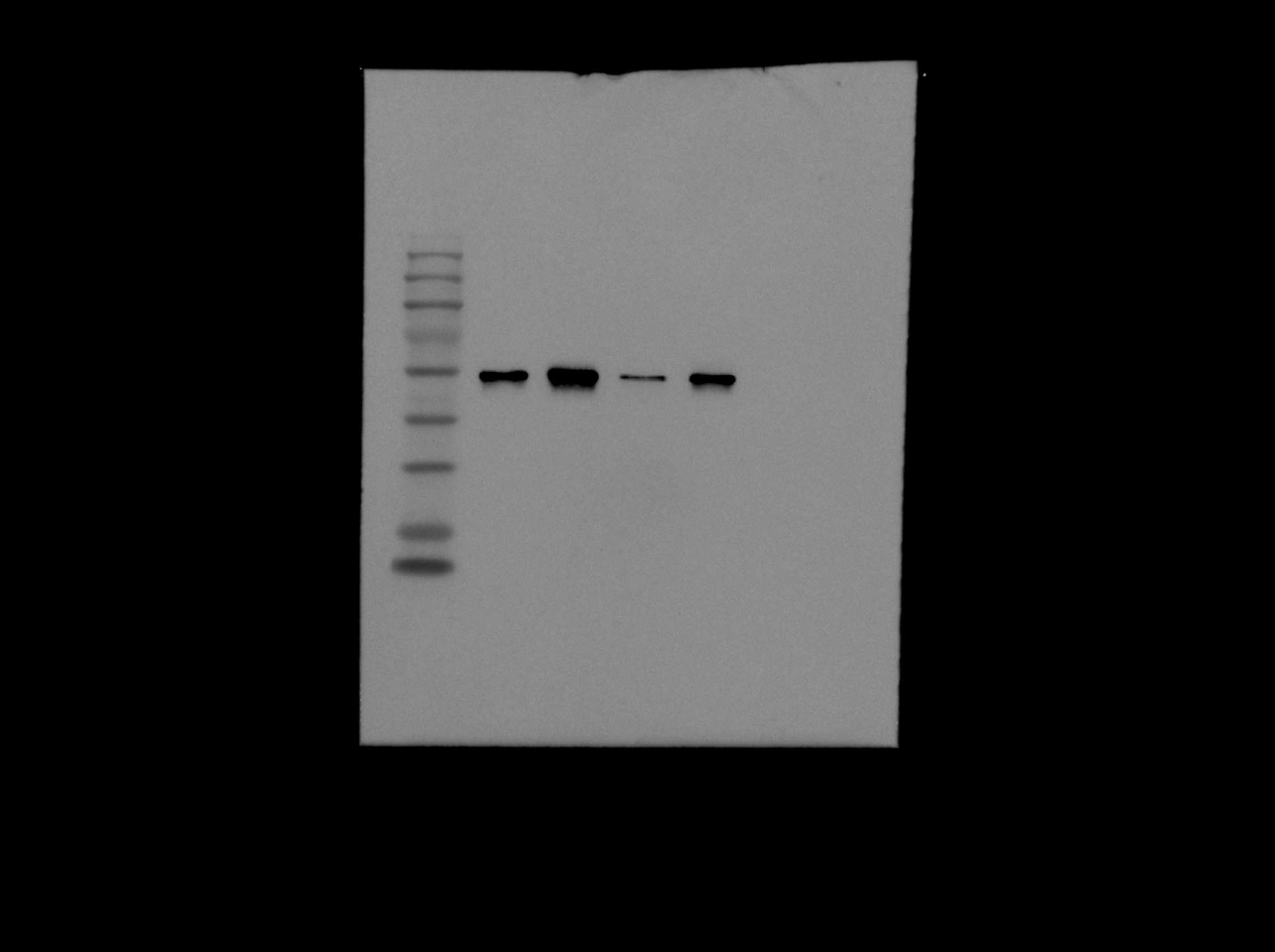


Figure2C-1


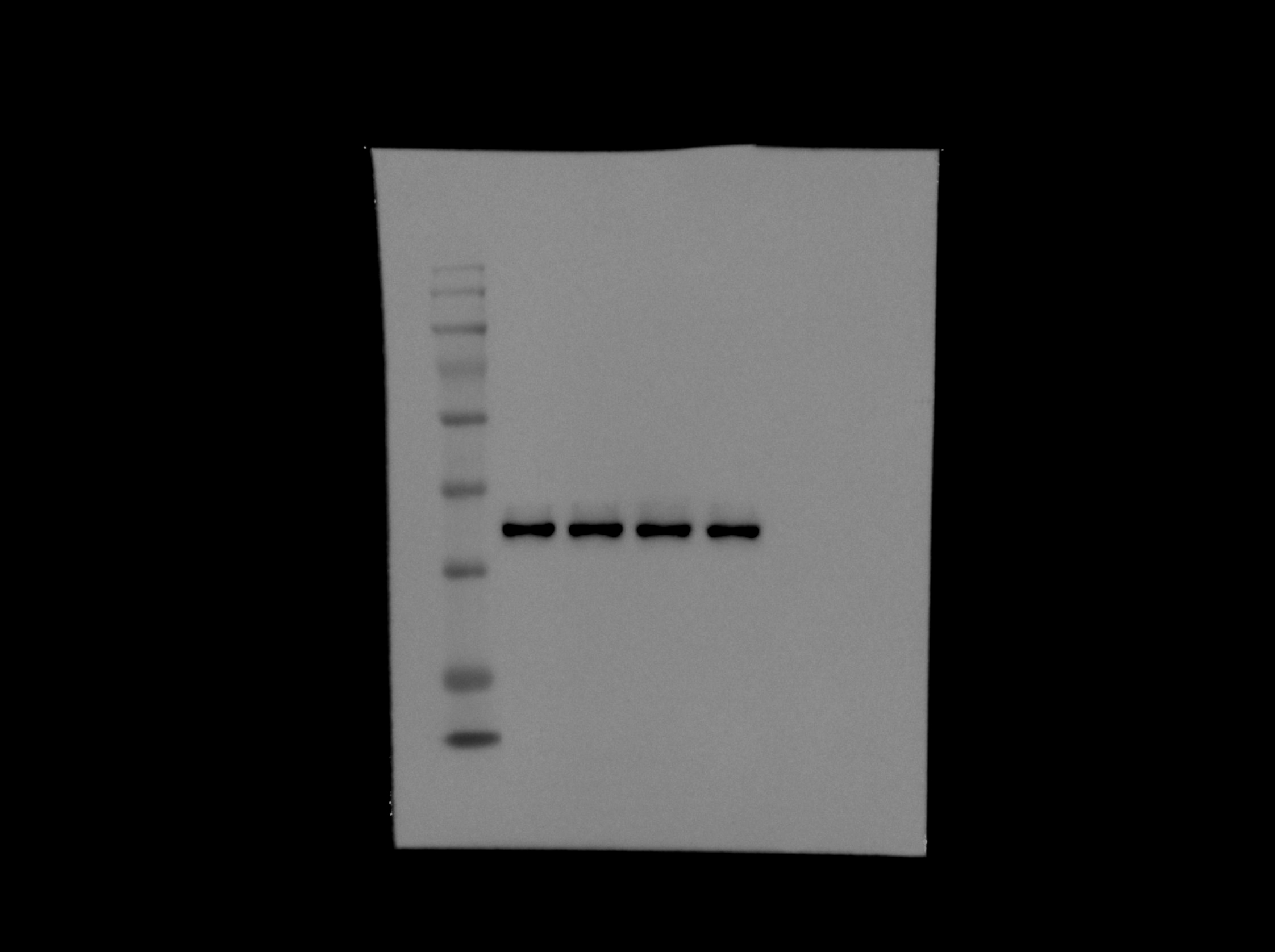


Figure2C-2


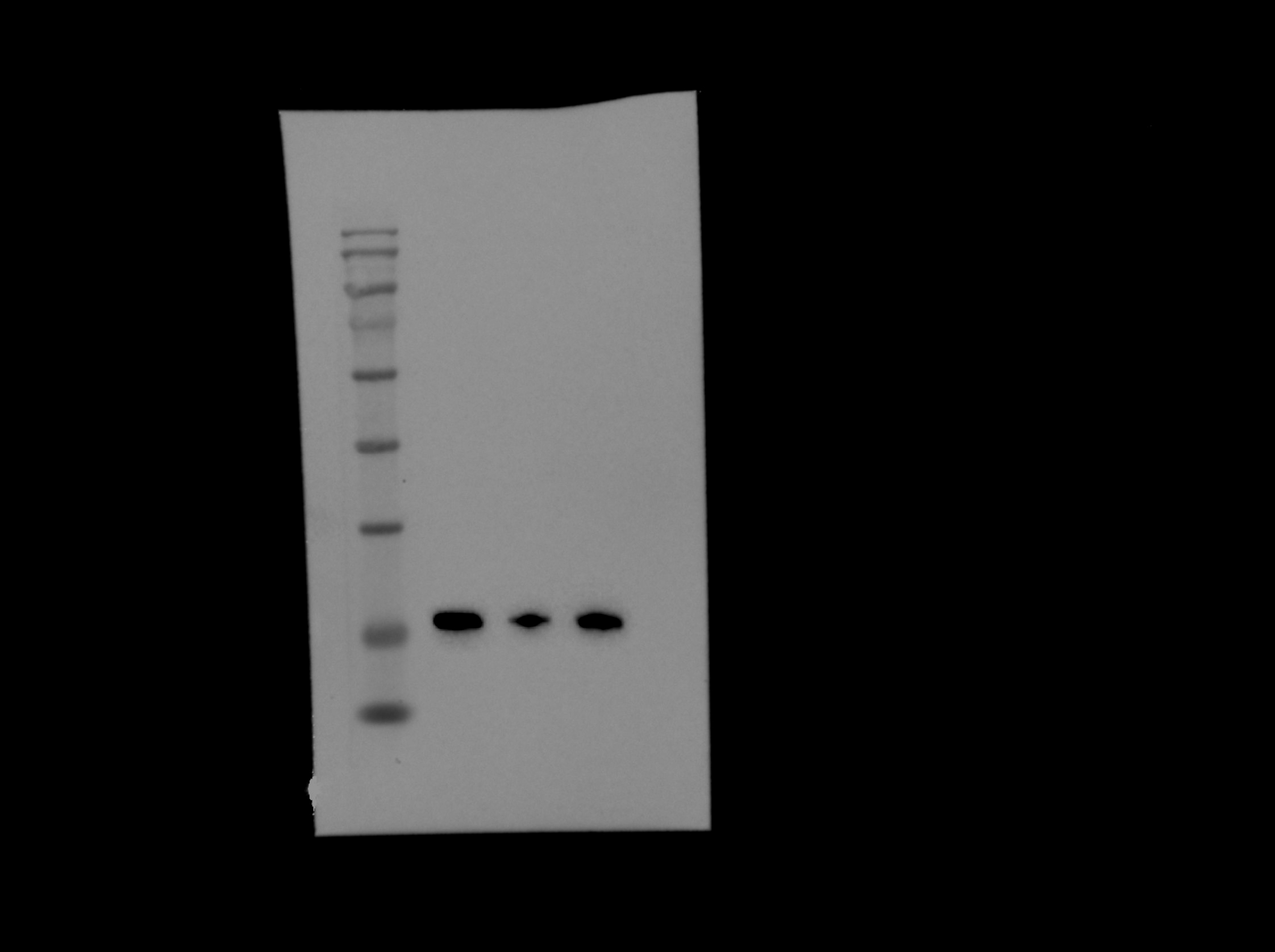


Figure3A-1


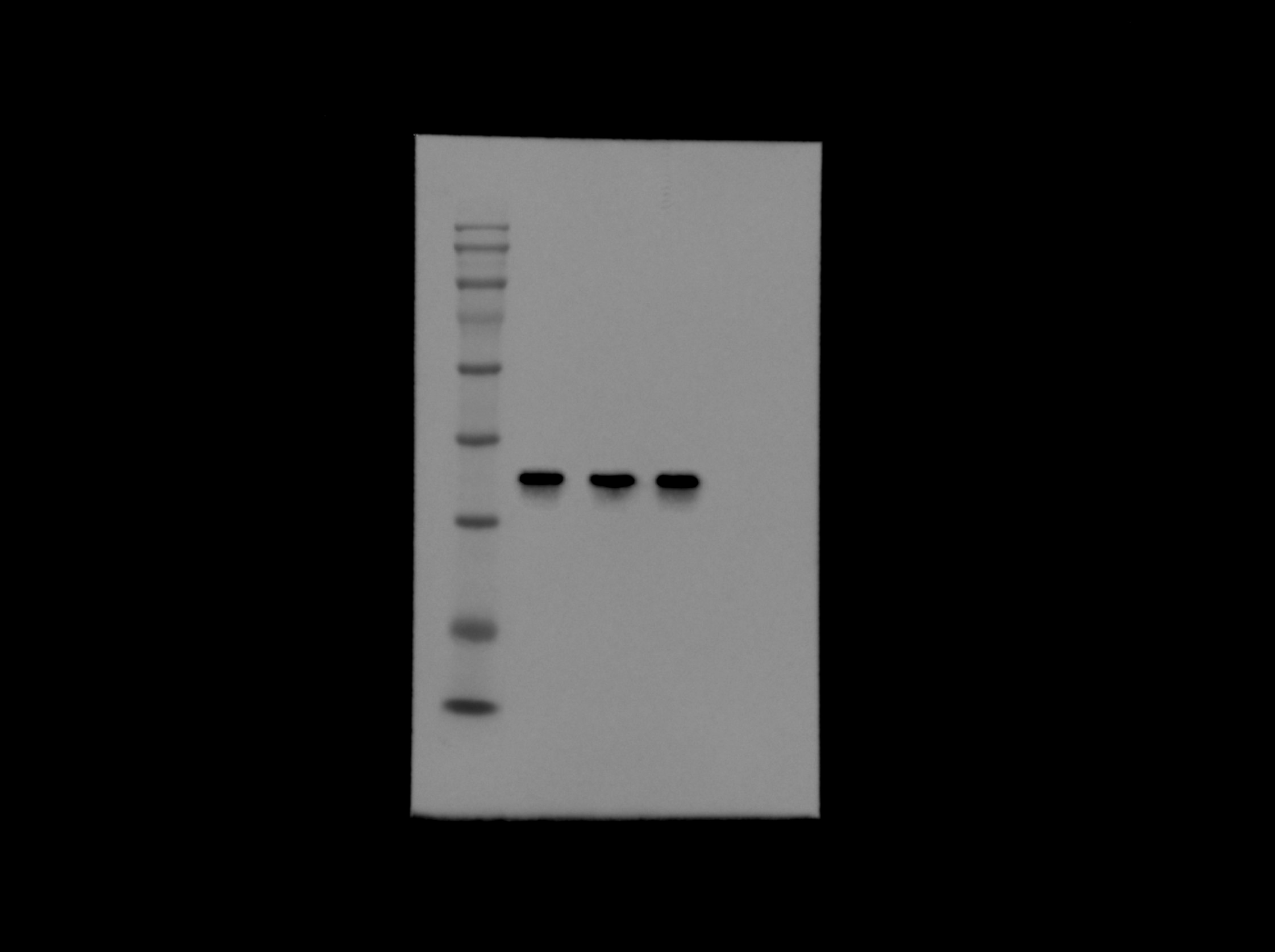


Figure3A-2


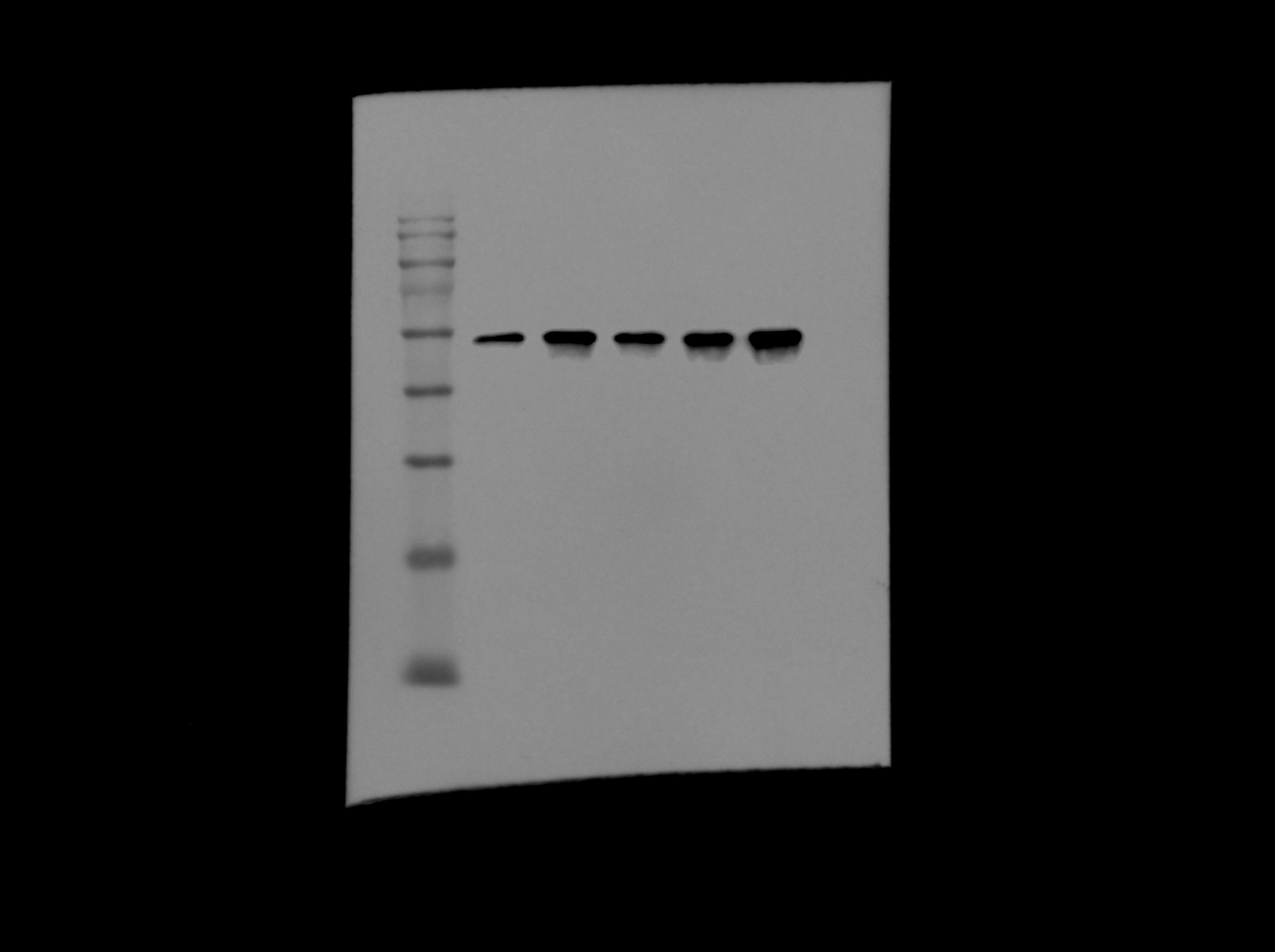


Figure3B-1


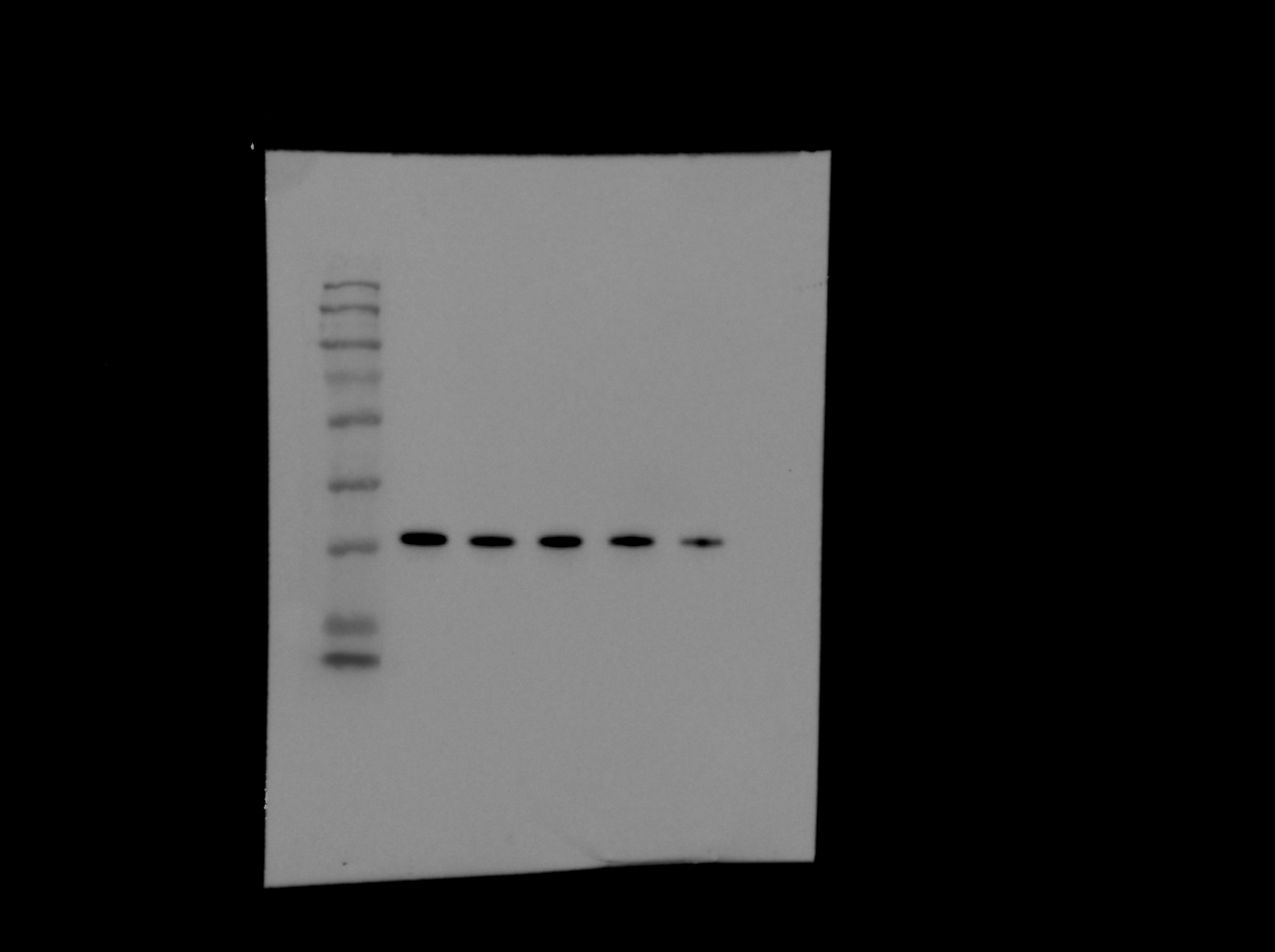


Figure3B-2


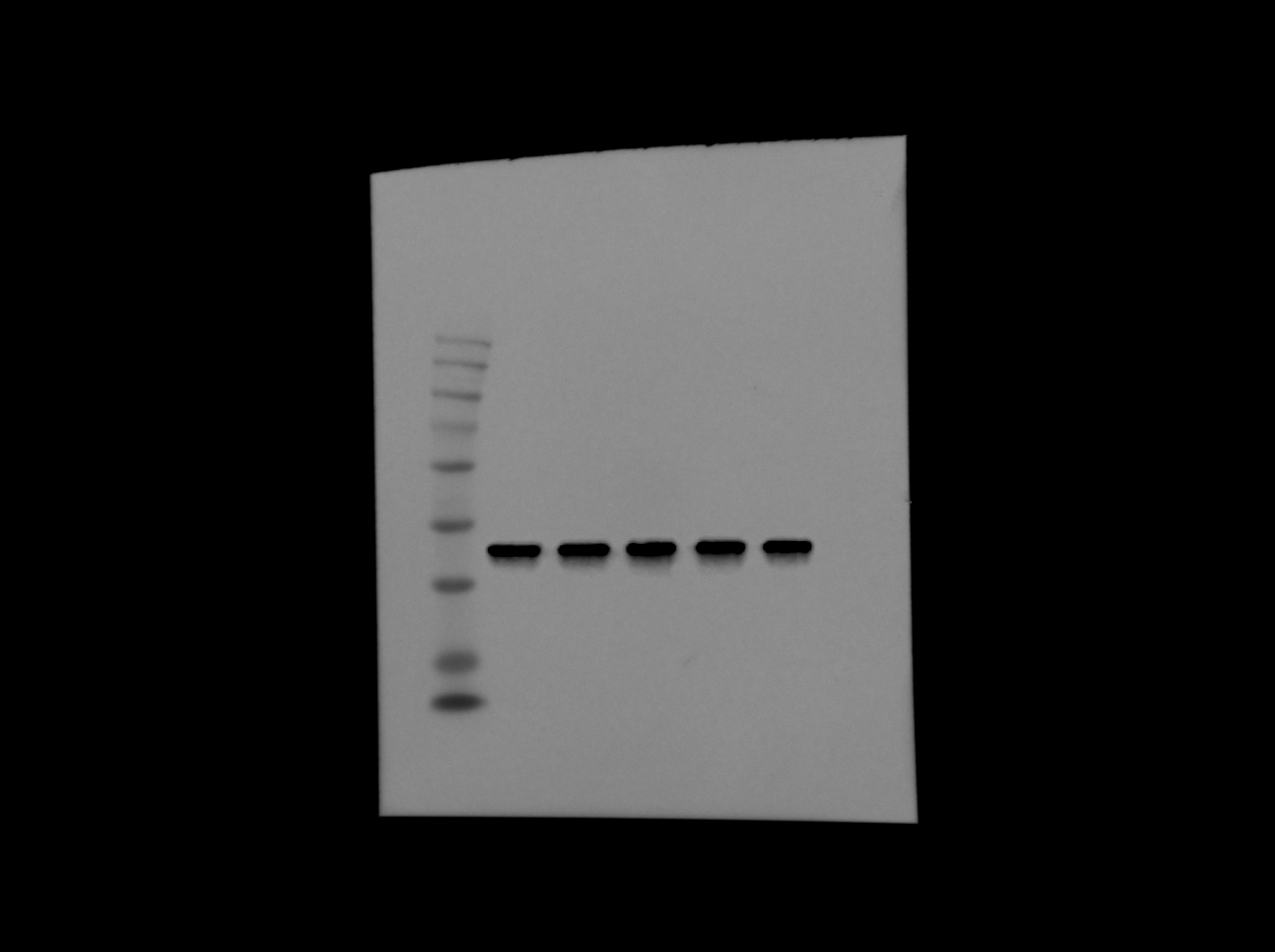


Figure3B-3


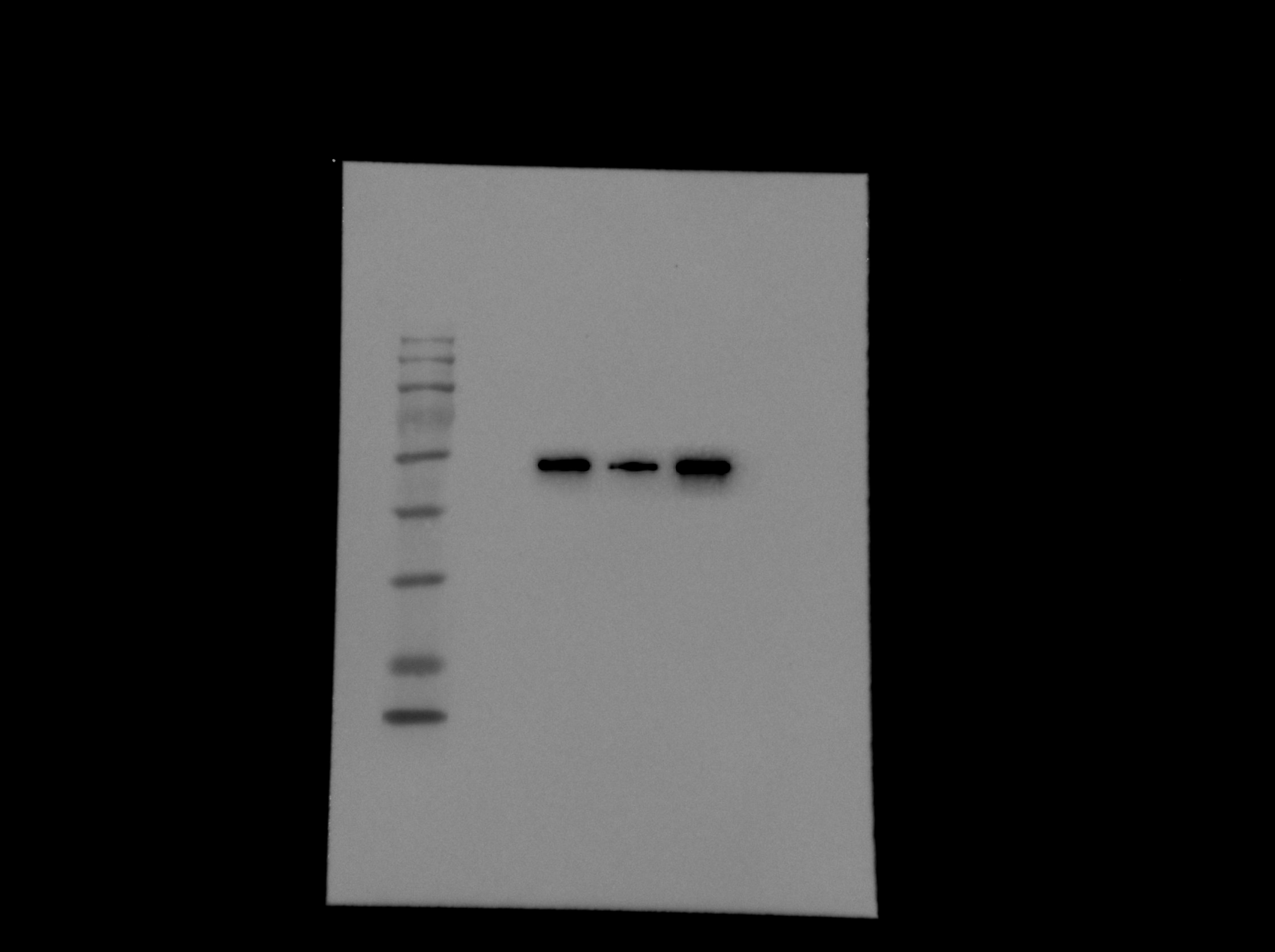


Figure3C-1


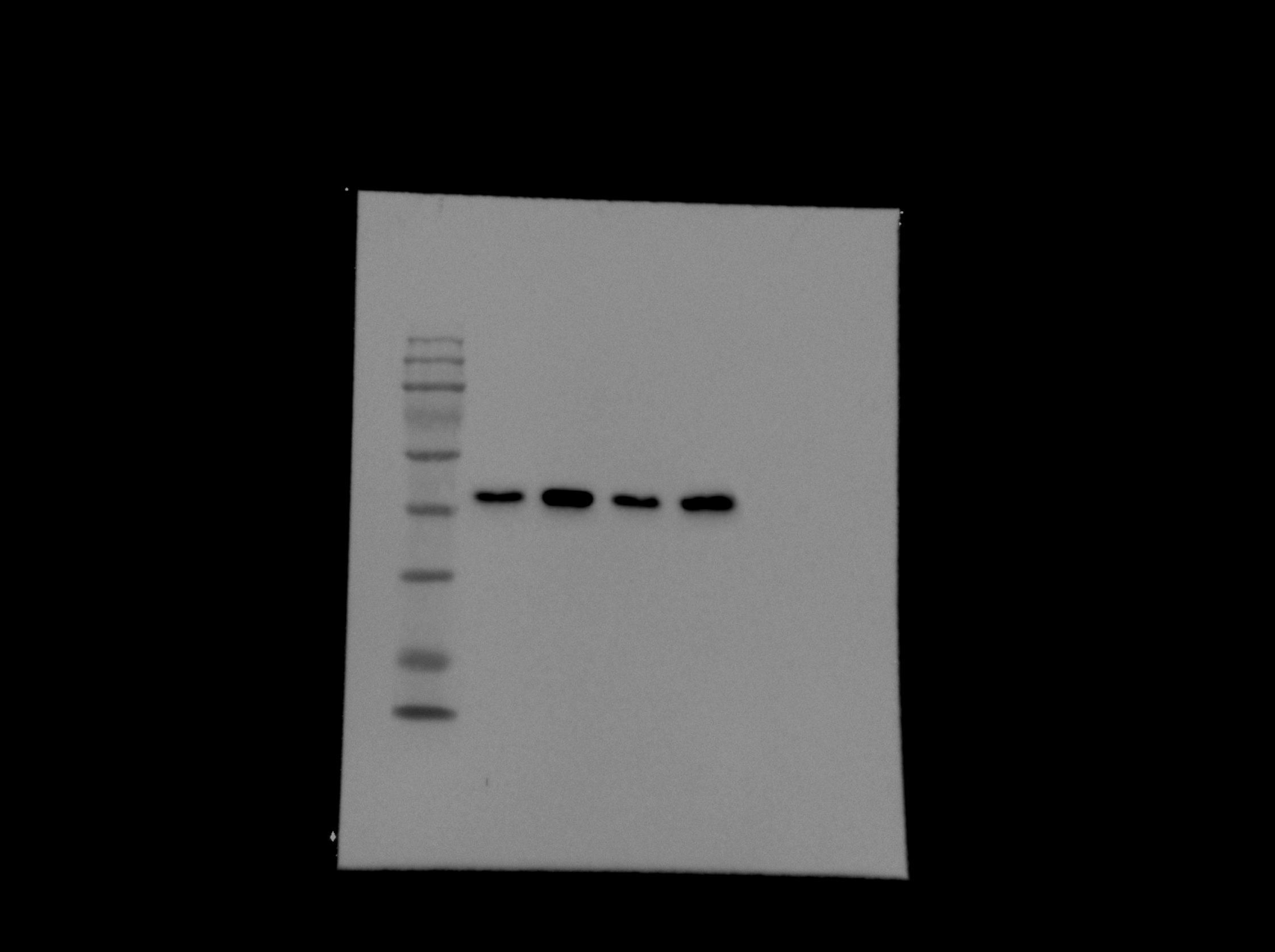


Figure3C-2


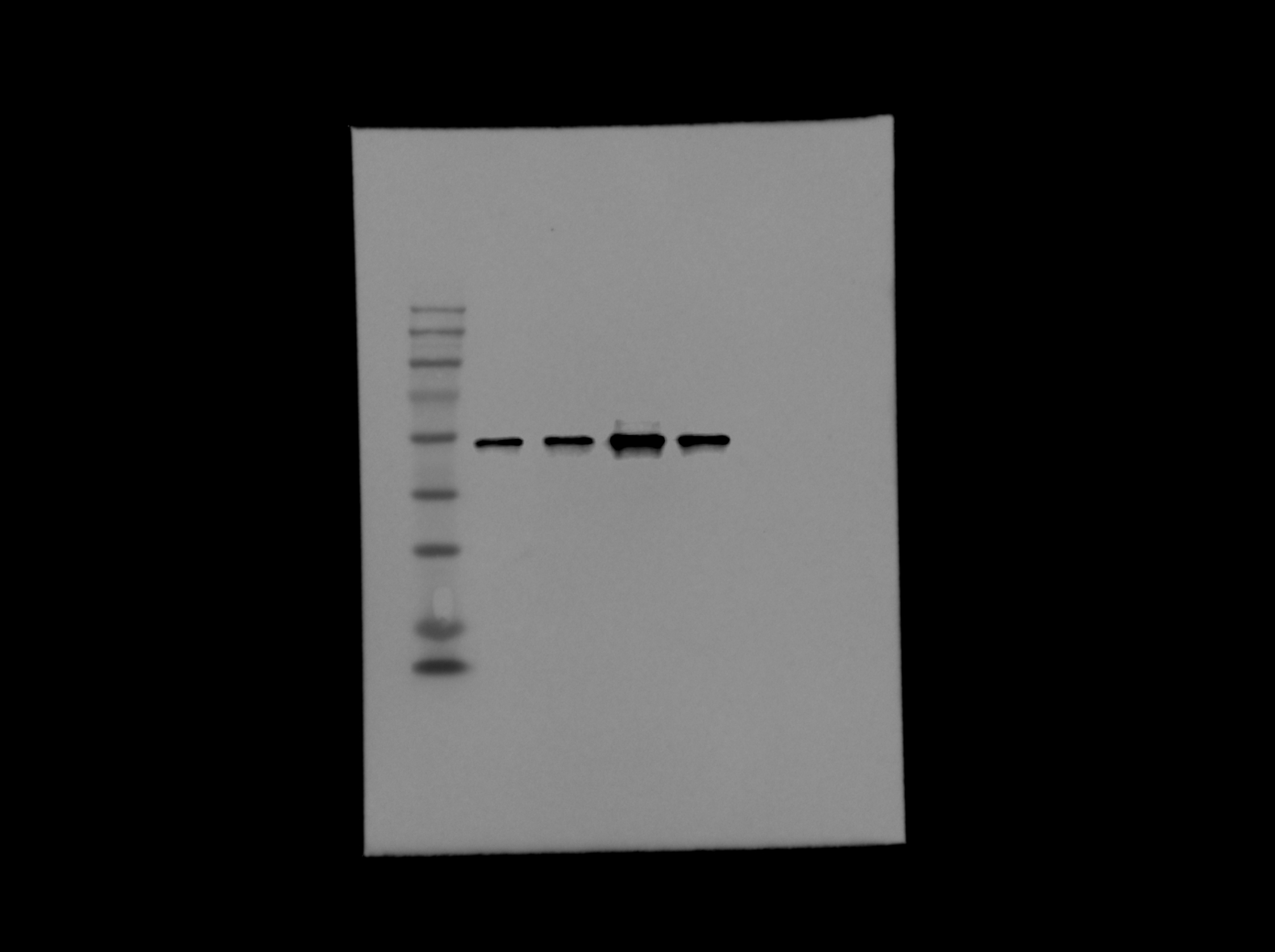


Figure3C-3


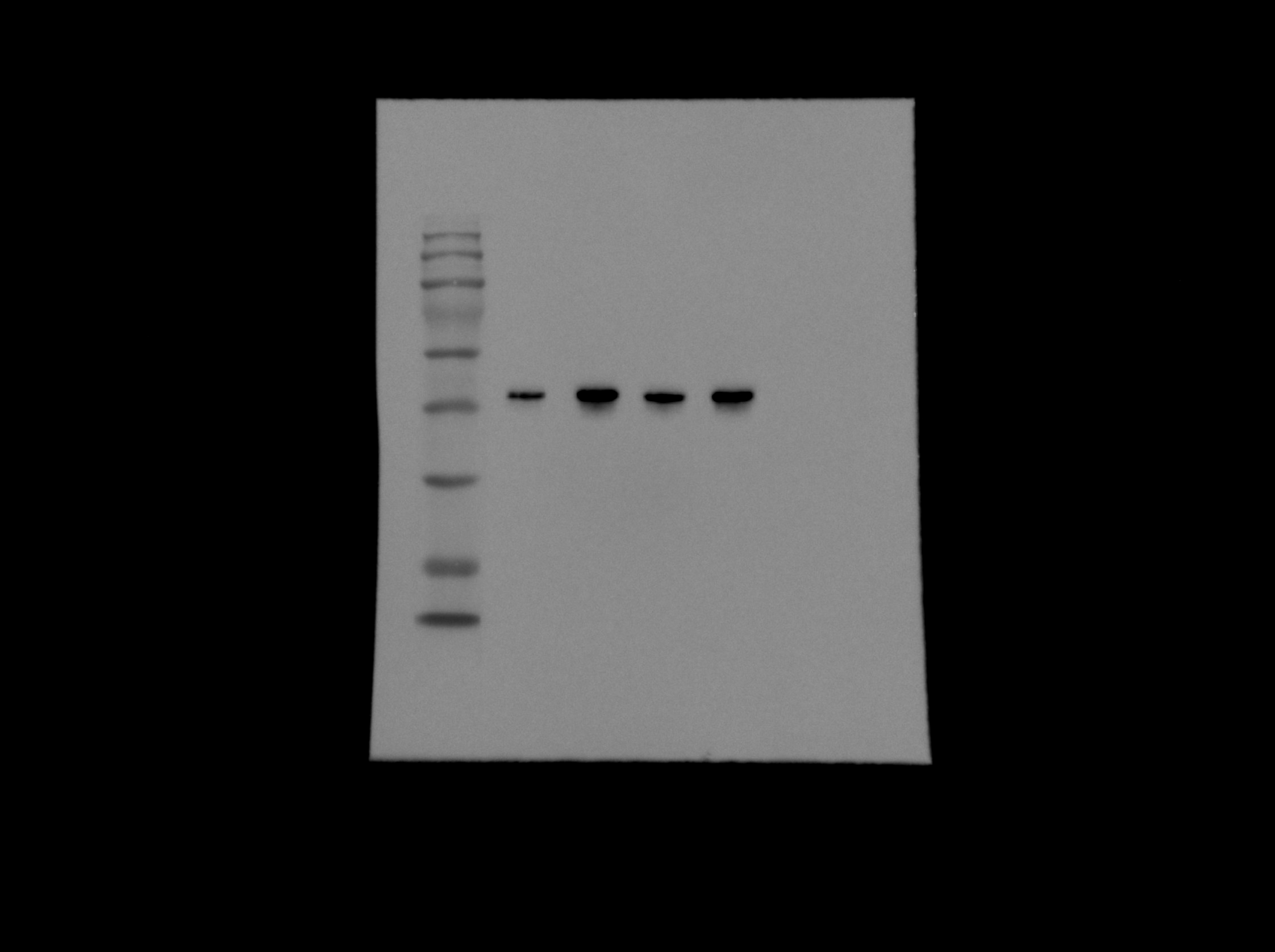


Figure3C-4


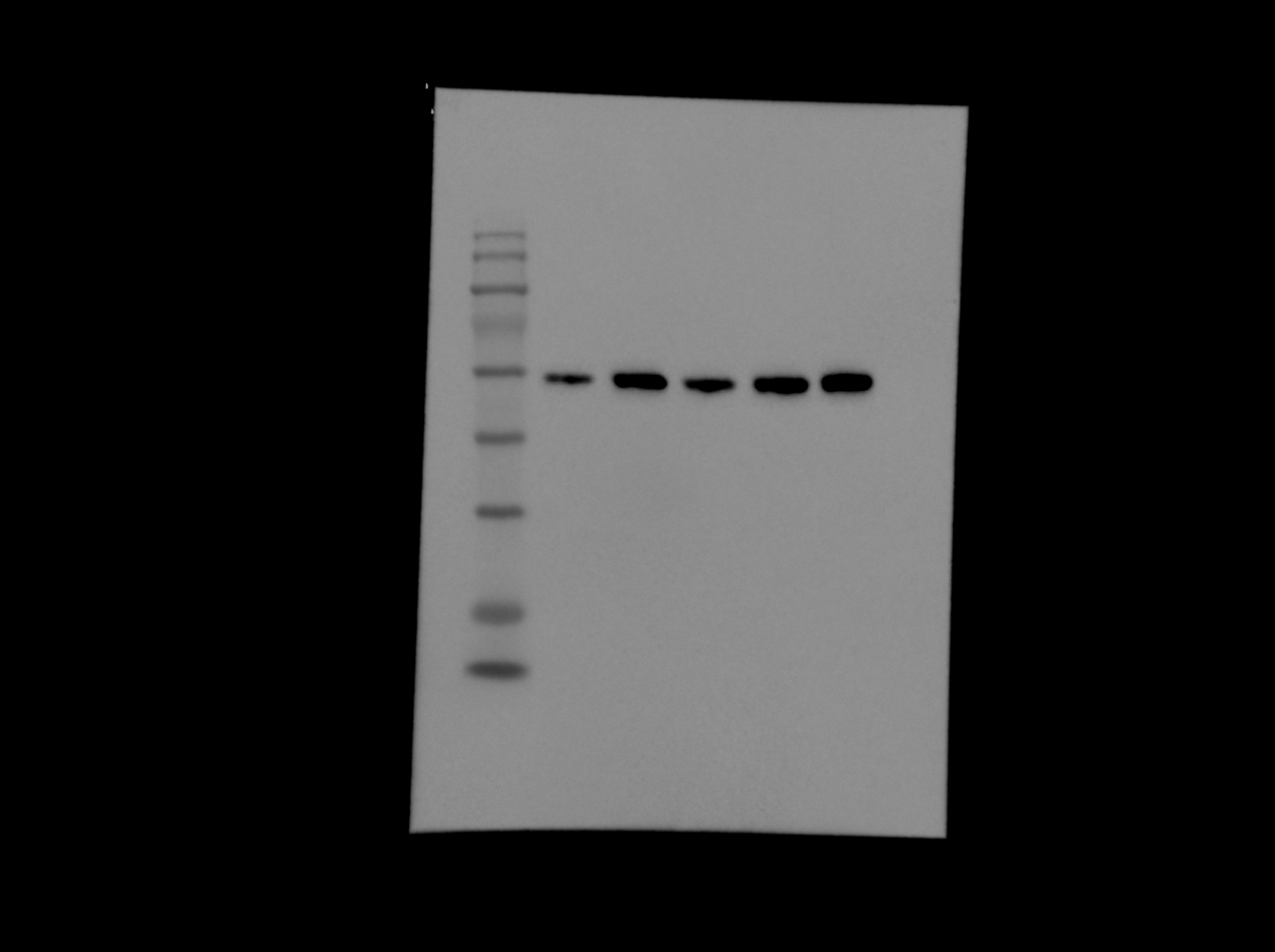


Figure3D-1


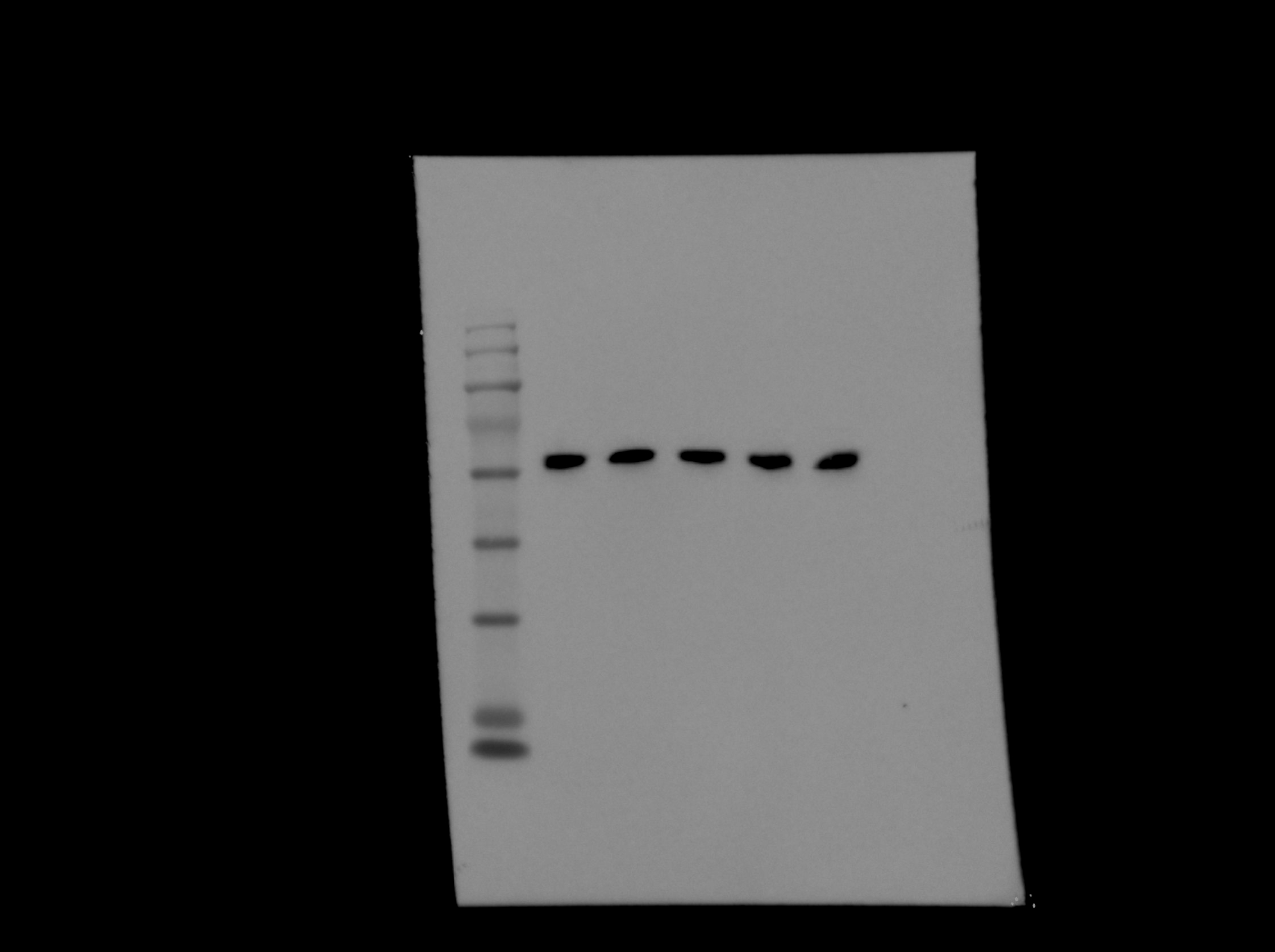


Figure3D-2


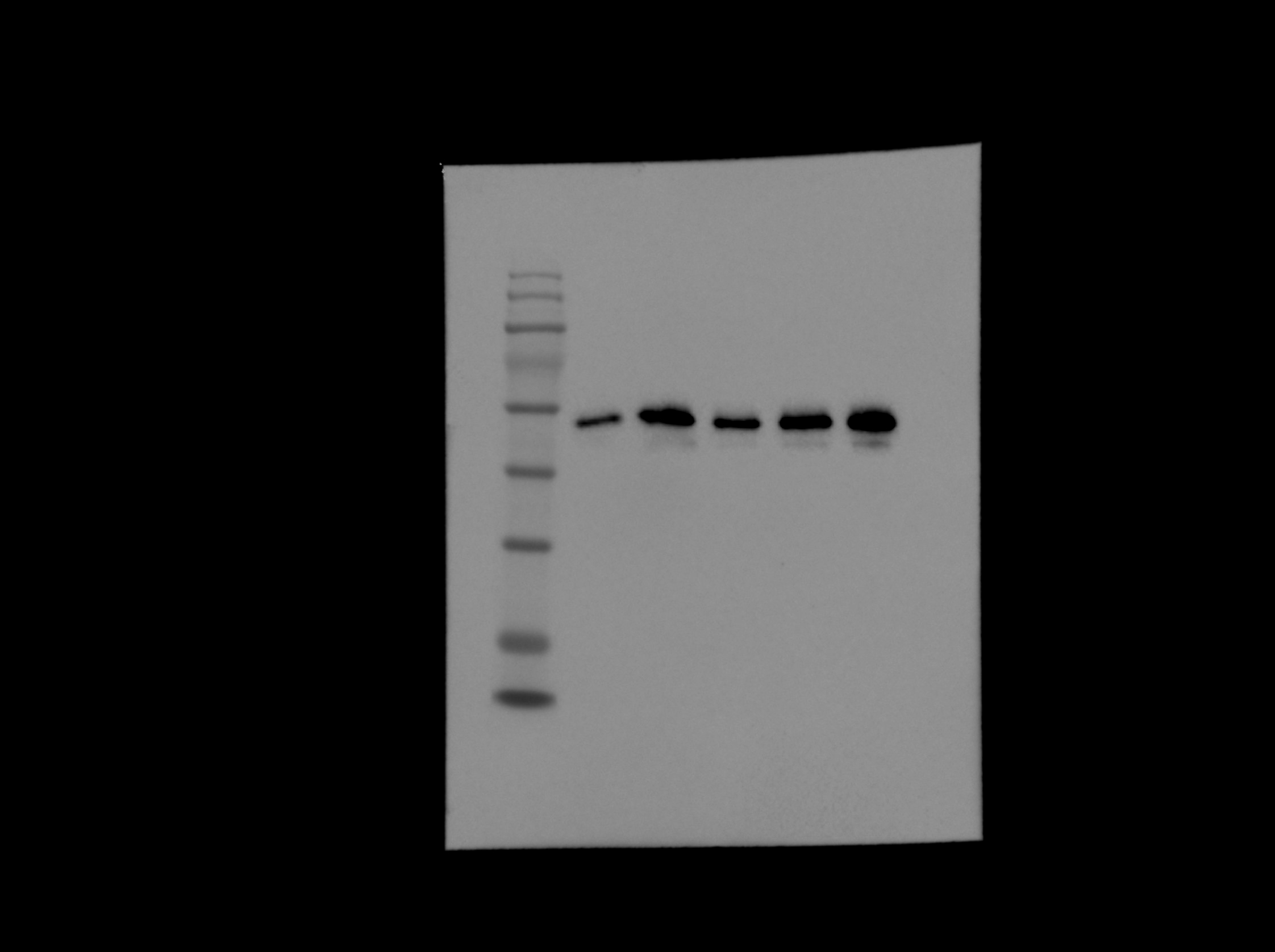


Figure3D-3


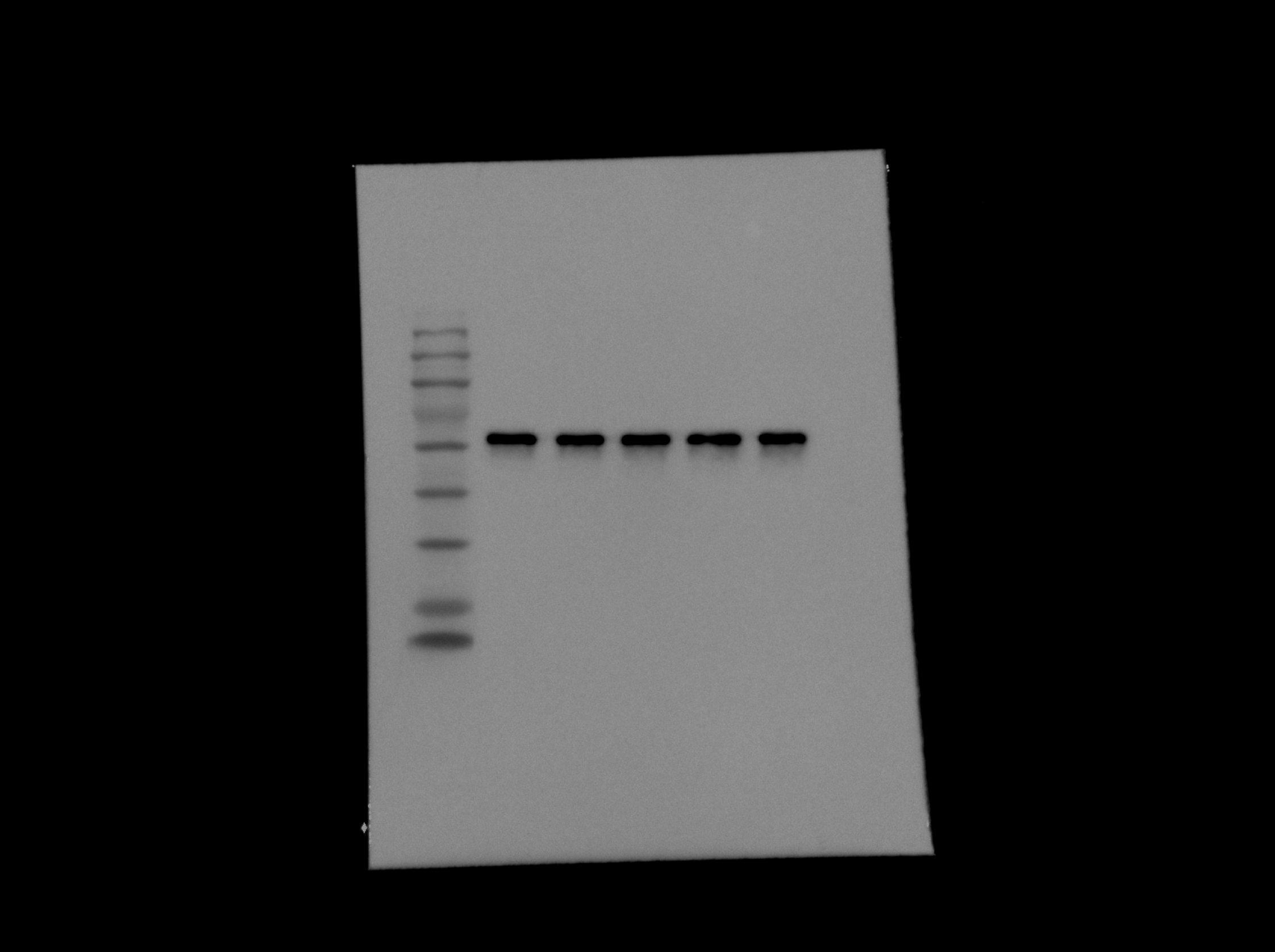


Figure3D-4


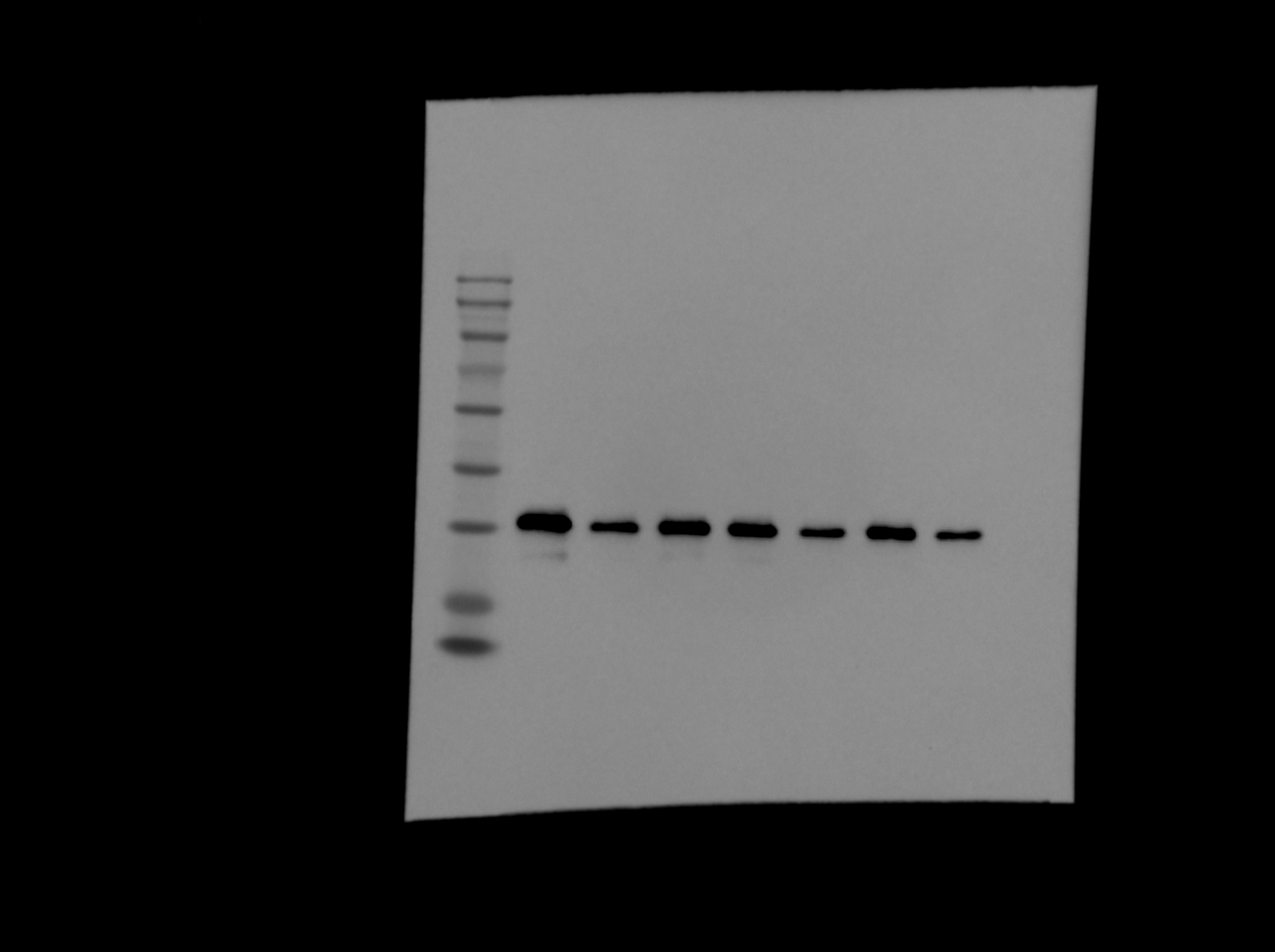


Figure5E-1


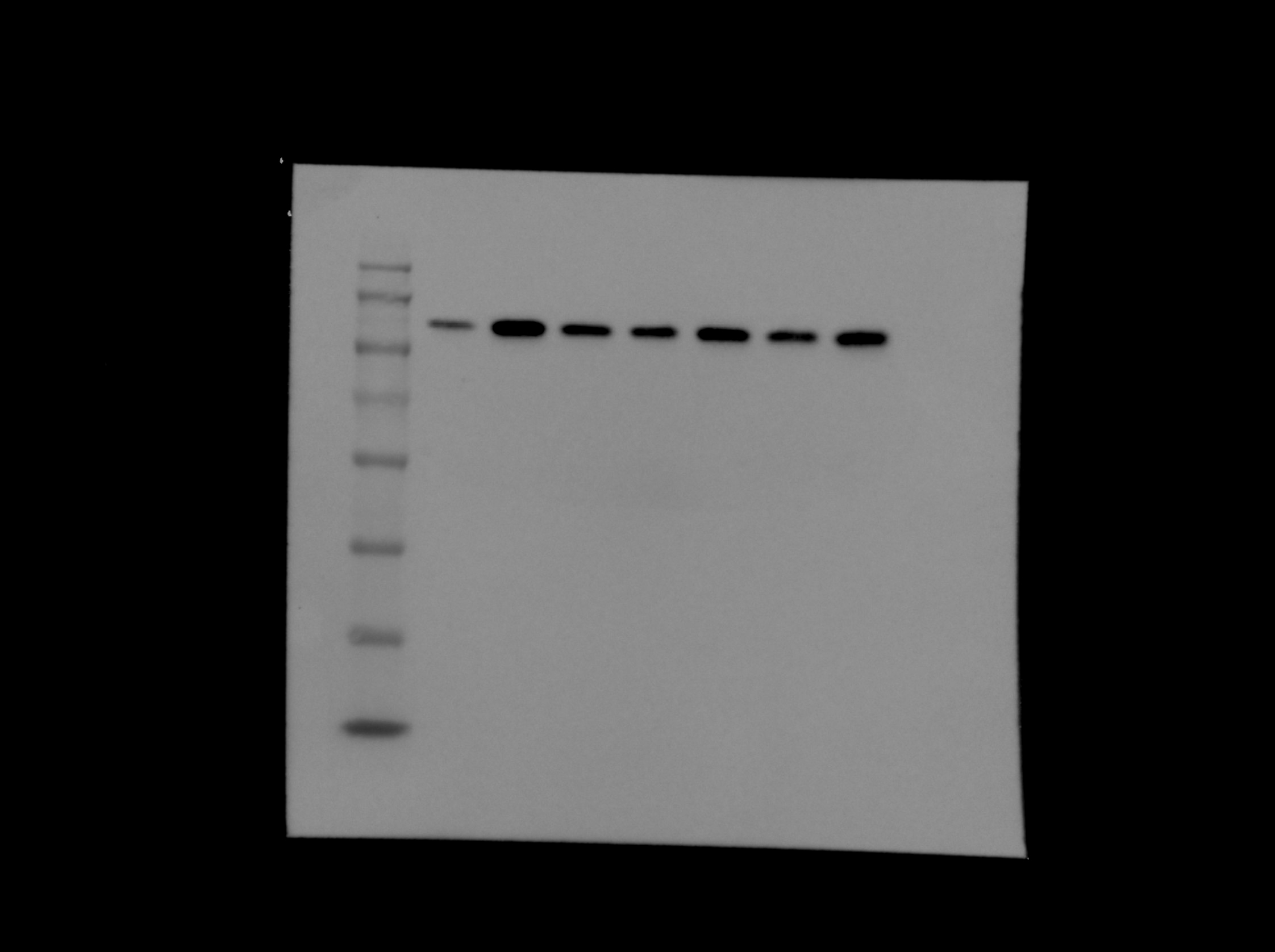


Figure5E-2


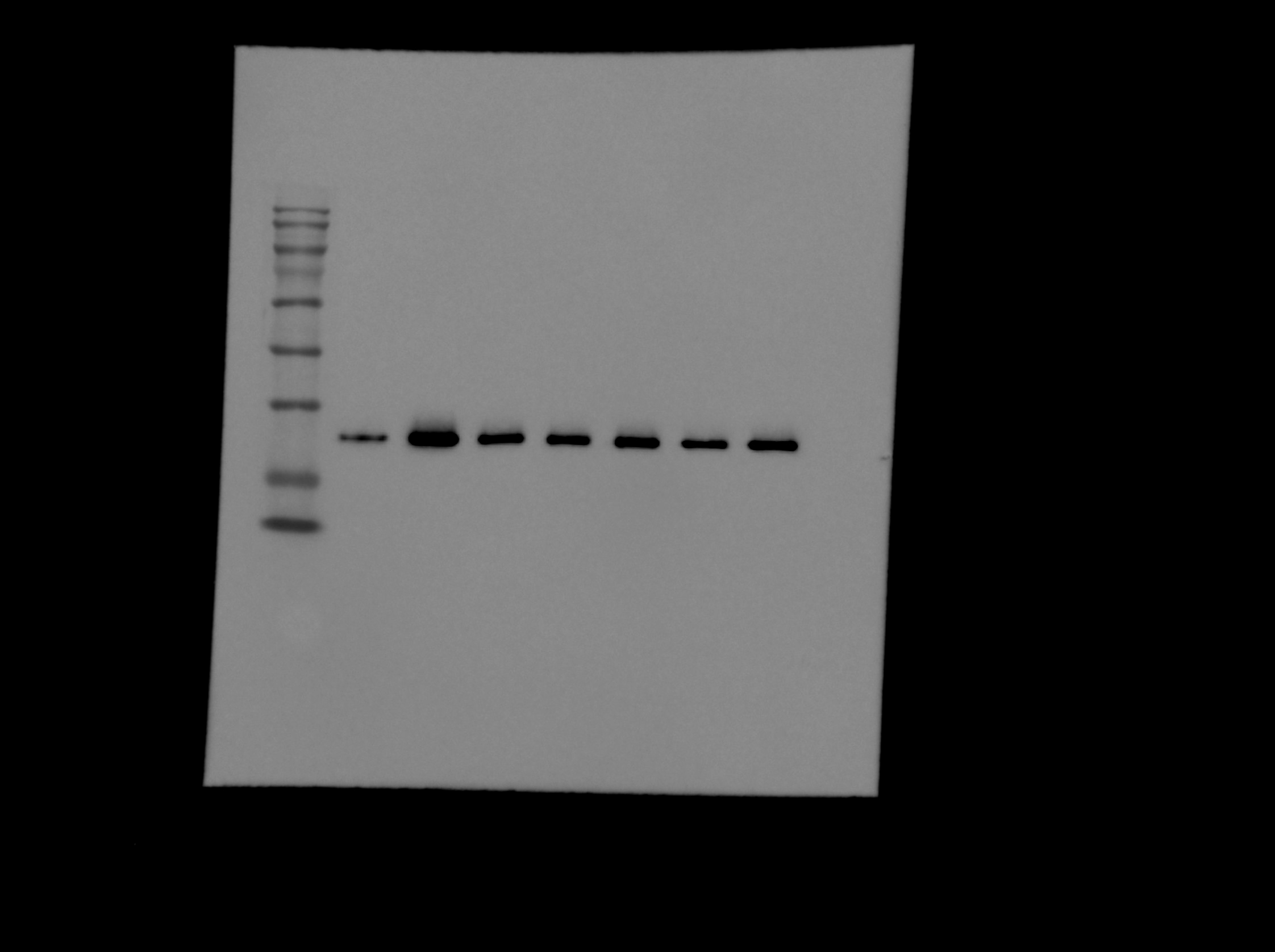


Figure5E-3


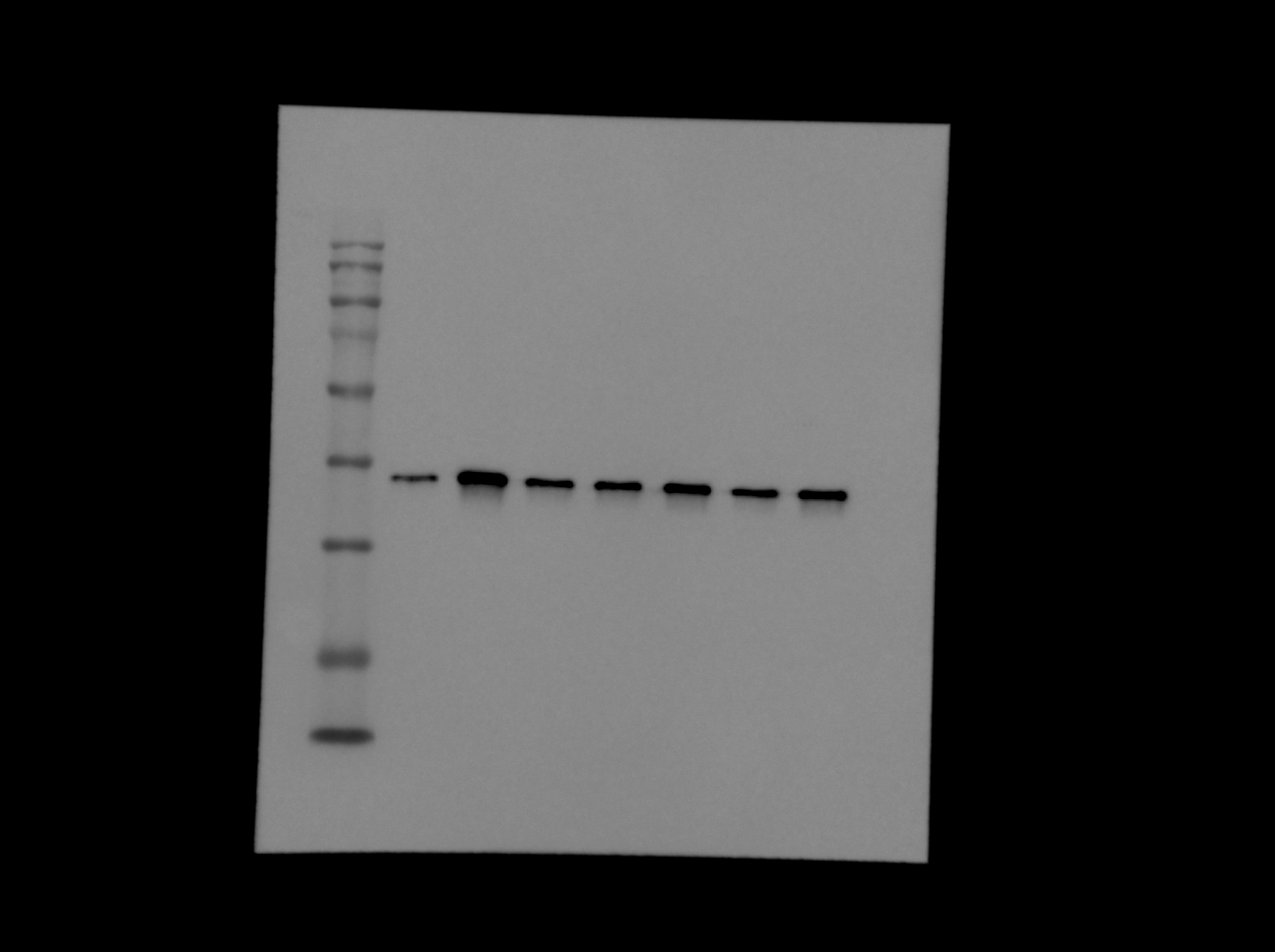


Figure5E-4


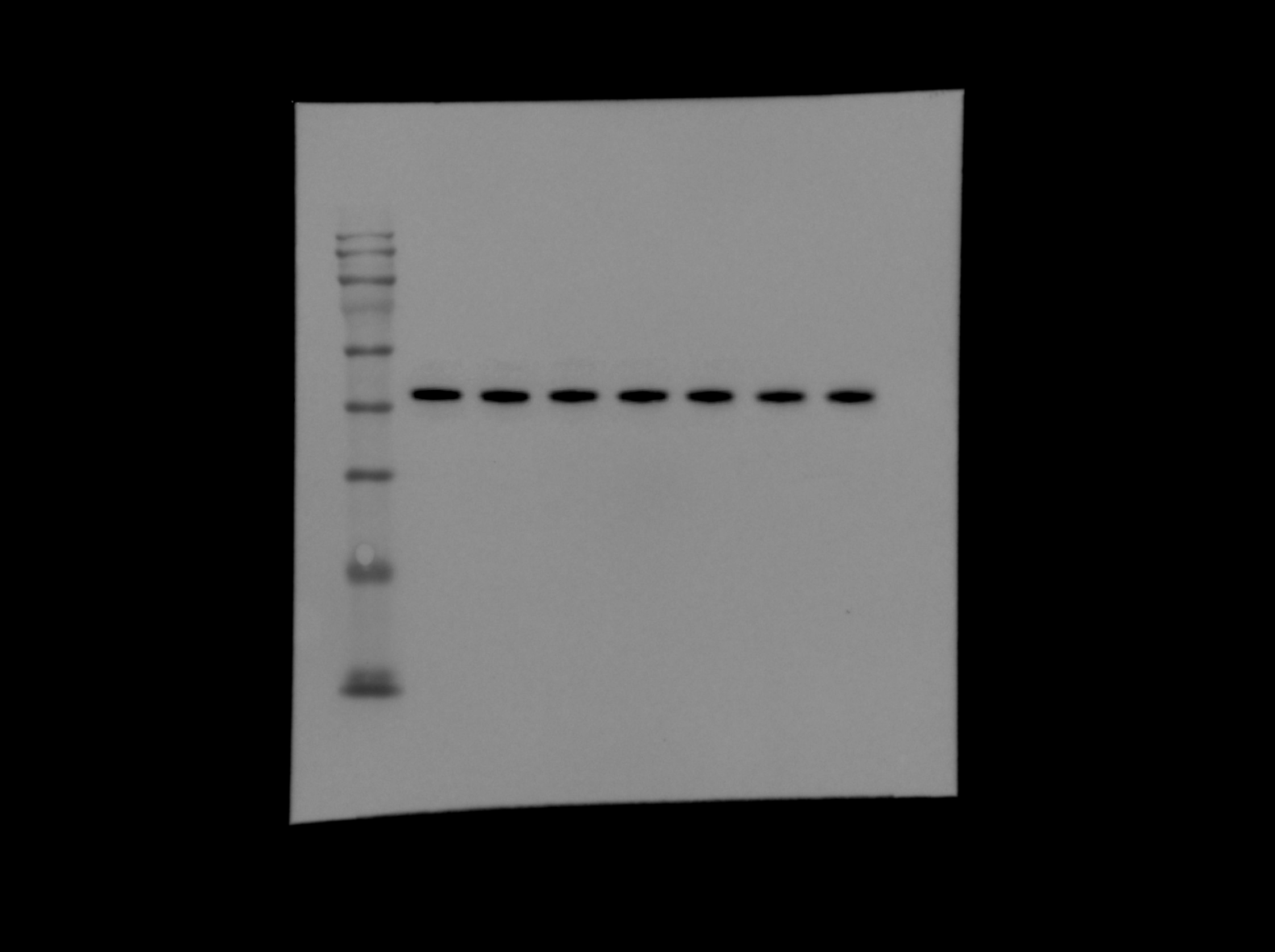


Figure5E-5
